# Structural evidence for the critical role of the prion protein hydrophobic region in forming an infectious prion

**DOI:** 10.1371/journal.ppat.1008139

**Published:** 2019-12-09

**Authors:** Romany Abskharon, Fei Wang, Alexandre Wohlkonig, Juxin Ruan, Sameh Soror, Gabriele Giachin, Els Pardon, Wenquan Zou, Giuseppe Legname, Jiyan Ma, Jan Steyaert

**Affiliations:** 1 Structural Biology Brussels, Vrije Universiteit Brussel (VUB), Brussels, Belgium; 2 VIB-VUB Center for Structural Biology, Vlaams Instituut Biotechnologie (VIB), Brussels, Belgium; 3 Center for Neurodegenerative Science, Van Andel Institute, Grand Rapids, Michigan, United States of America; 4 National Institute of Oceanography and Fisheries (NIOF), Cairo, Egypt; 5 Center of Excellence, Helwan Structural Biology Research, Faculty of Pharmacy, Helwan University, Cairo, Egypt; 6 Structural Biology Group, European Synchrotron Radiation Facility, Grenoble, France; 7 Departments of Pathology and Neurology, Case Western Reserve University School of Medicine, Cleveland, Ohio, United States of America; 8 Laboratory of Prion Biology, Department of Neuroscience, Scuola Internazionale Superiore di Studi Avanzati (SISSA), Trieste, Italy; Dartmouth College Geisel School of Medicine, UNITED STATES

## Abstract

Prion or PrP^Sc^ is the proteinaceous infectious agent causing prion diseases in various mammalian species. Despite decades of research, the structural basis for PrP^Sc^ formation and prion infectivity remains elusive. To understand the role of the hydrophobic region in forming infectious prion at the molecular level, we report X-ray crystal structures of mouse (Mo) prion protein (PrP) (residues 89–230) in complex with a nanobody (Nb484). Using the recombinant prion propagation system, we show that the binding of Nb484 to the hydrophobic region of MoPrP efficiently inhibits the propagation of proteinase K resistant PrP^Sc^ and prion infectivity. In addition, when added to cultured mouse brain slices in high concentrations, Nb484 exhibits no neurotoxicity, which is drastically different from other neurotoxic anti-PrP antibodies, suggesting that the Nb484 can be a potential therapeutic agent against prion disease. In summary, our data provides the first structure-function evidence supporting a crucial role of the hydrophobic region of PrP in forming an infectious prion.

## Author summary

Prion disease is caused by the misfolding of normal prion protein (PrP^C^) to its pathogenic isoform, termed PrP^Sc^. The underlying mechanism for such fatal misfolding is still unknown, which greatly impedes the development of efficacious therapeutic strategies against these currently incurable diseases. Previously, we reported the crystal structure of human PrP in complex with a nanobody (Nb484), which could inhibit mouse prion propagation in a cell culture model. To understand the molecular mechanism for the inhibitory action of Nb484, in this study, firstly we determine the crystal structures of mouse PrP-Nb484 complexes and show that Nb484 binds to and stabilizes the hydrophobic region of PrP. Then in our newly developed recombinant prion propagation system, we show that Nb484 inhibits prion propagation through the competitive inhibition mechanism. We further demonstrate that, contrary to certain neurotoxic anti-PrP antibodies, the inhibitory Nb484 exhibits no neurotoxicity on cultured organotypic brain slices, indicating the great therapeutic potential of Nb484. In summary, our current study provides the first structure-function evidence supporting a critical role of the hydrophobic region in forming the infectious prion and highlights the therapeutic potential of Nb484.

## Introduction

Prion diseases, also known as transmissible spongiform encephalopathies (TSEs), are a group of fatal neurodegenerative diseases affecting both humans and animals[1]. The prion protein (PrP) exists in two forms: the physiological cellular isoform, PrP^C^, and the disease-associated infectious isoform, denoted as prion or PrP^Sc^[2]. The host-encoded PrP^C^ is a cell surface glycosylphosphatidylinositol (GPI)-anchored glycoprotein containing a flexible N-terminal fragment and a well-folded α-helical C-terminus. PrP^Sc^, however, is composed almost entirely of β-sheets[3]. The conversion from normal PrP^C^ to prion is the underlying pathogenic event of prion diseases[4]. Detailed three-dimensional (3D) structures of different mammalian PrP^C^ have been solved[5–7], but little atomic-level information is available on the PrP^Sc^ structure except for a recent study that proposes the 4-rung β-solenoid architecture of PrP^Sc^[8]. Thus far, the molecular mechanisms of the conformational conversion of PrP^C^ into infectious prions and other key neurodegenerative processes in prion diseases remain unclear, which is a road block for developing effective therapeutic strategies against these devastating neurodegenerative disorders[8].

Previous structural studies have established that PrP^C^ features an intrinsically unstructured N-terminal region, a globular C-terminal domain containing three α-helices and two short β-strands—forming a unique β1-α1- β2- α2- α3 fold in which the β-strands come together to form an anti-parallel β-ribbon (SCOP classification) and a highly conserved middle region that links the flexible N-terminus and globular C-terminus. This conserved middle region consists of a cluster of four positively-charged lysine residues (101, 104, 106 and 110, human numbering) and a hydrophobic region (residues 112–135, human numbering), which has a high propensity for β-sheet secondary structure[9, 10]. Therefore, the hydrophobic region has been proposed to be involved in PrP conformational changes or prion propagation, which occurs when PrP^Sc^ acts as a template and induces the seeded conformational change of PrP^C^ into PrP^Sc^, and causes prion disease.

A large number of anti-PrP antibodies have been generated. Interestingly, it has been shown that anti-PrP monoclonal antibodies are able to inhibit prion propagation *in vitro* and *in vivo*, presumably by stabilizing the PrP^C^ conformation. Therefore, passive immunization with anti-PrP antibodies presents a promising therapeutic approach against TSEs[11]. We recently reported that an anti-PrP camelid heavy chain antibody or nanobody (Nb), namely Nb484, inhibits prion conversion in cultured mouse neuronal cells in a dose-dependent manner[12].

To understand the structural basis of Nb484 mediated inhibition, here we report two new X-ray crystal structures of mouse (Mo) PrP•Nb484 complexes at pH 6.0 and pH 8.0 resolved with a resolution of 2.1 Å and 1.2 Å, respectively; and the crystal structure of the nanobody Nb484 alone (at 1.2 Å resolution). Similar to a previous report on the human prion protein[12], our current study reveals that Nb484 allows the crystallization of MoPrP polypeptide from residues 118 to 226 and that the hydrophobic region folds into a stable three-stranded antiparallel β-sheet arrangement. In both structures at different pH, Nb484 stabilizes residues 120–122 of MoPrP into a β-strand, termed β0, which folds into a three-stranded antiparallel β-sheet with β1 and β2. We further demonstrate the prion inhibitory effect of Nb484 on the mouse PrP in Protein Misfolding Cyclic Amplification (PMCA) reactions. Together, our study provides convincing structure-function evidence for the critical role of the hydrophobic region in converting normal PrP^C^ to pathogenic PrP^Sc^.

## Results

### Crystal structures of MoPrP•Nb484 complexes

Using a nanobody as a crystallization chaperone, we obtained well diffracting crystals of MoPrP(89–230) in complex with Nb484 at different pH. In these complexes, the total amount of structured polypeptide (125 amino acids of antibody and 108 amino acids of the prion protein) rises to 71% in contrast to 52% for free PrP, thus providing a much better starting point for crystallization. The first high-resolution MoPrP(89–230)•Nb484 crystal was obtained at pH 6.0, and the structure was refined to 2.1 Å resolution. The second complex was crystallized at pH 8.0 and determined to 1.2 Å resolution. Both structures contain residues from 118–226 and the average backbone root-mean-square distance between them is only 0.609 Å, indicating that both structures are almost identical. X-ray data-collection and crystal structure-refinement statistics are summarized in Table 1.

**Table 1:** Statistics of X-ray diffraction data collection and atomic refinement of MoPrP•Nb484 complexes and free Nb484.

	Crystal 1, MoPrP(89–230)•Nb484 at pH 8.0	Crystal2, MoPrP(89–230)•Nb484 at pH 6.0	Nb484
**Data collection**			
Space group	P212121	P1 21 1	P212121
**Cell dimension**			
*a*, *b*, *c* (Å)	37.32, 74.64, 116.47	59.13, 63.80, 69.79	30.4, 37.15, 83.00
a, b, g (°)	90,00 90.00, 90.00	90.00, 101.96, 90.00	90, 90, 90
Resolution (Å)	37.32 (1.199–1.230)	57.85 (2.10–2.22)	17.85 (1.230–1.258)
Rmerge (%)	24(49.6)	10.5 (80)	5.4 (19.4)
I/s(I) last shell	26.9(3.1)	10.3 (1.5)	11.8 (4.4)
Completeness (%)	93.59 (82.3)	99.5 (97.6)	98.8 (98.19)
No. reflections	96167	29594	26127
Rwork/Rfree	16.8/19.4	20.6/24.5	19.8/22.3
Solvent, %	75.36	44.6	25.68
No. all atoms	1982	3860	1024
Average B- Factor, Å	11.4	29.7	11.8
**r.m.s. deviations**			
Bond length (Å)	0.05	0.03	0.01
Bond angle (degrees)	0.781	0.55	1.3
Ramachandran plot (%)	99.5	98	99.2
Matthews coefficient, VM (Å3 Da-1)	4.99	2.22	1.67

**Fig 1:**
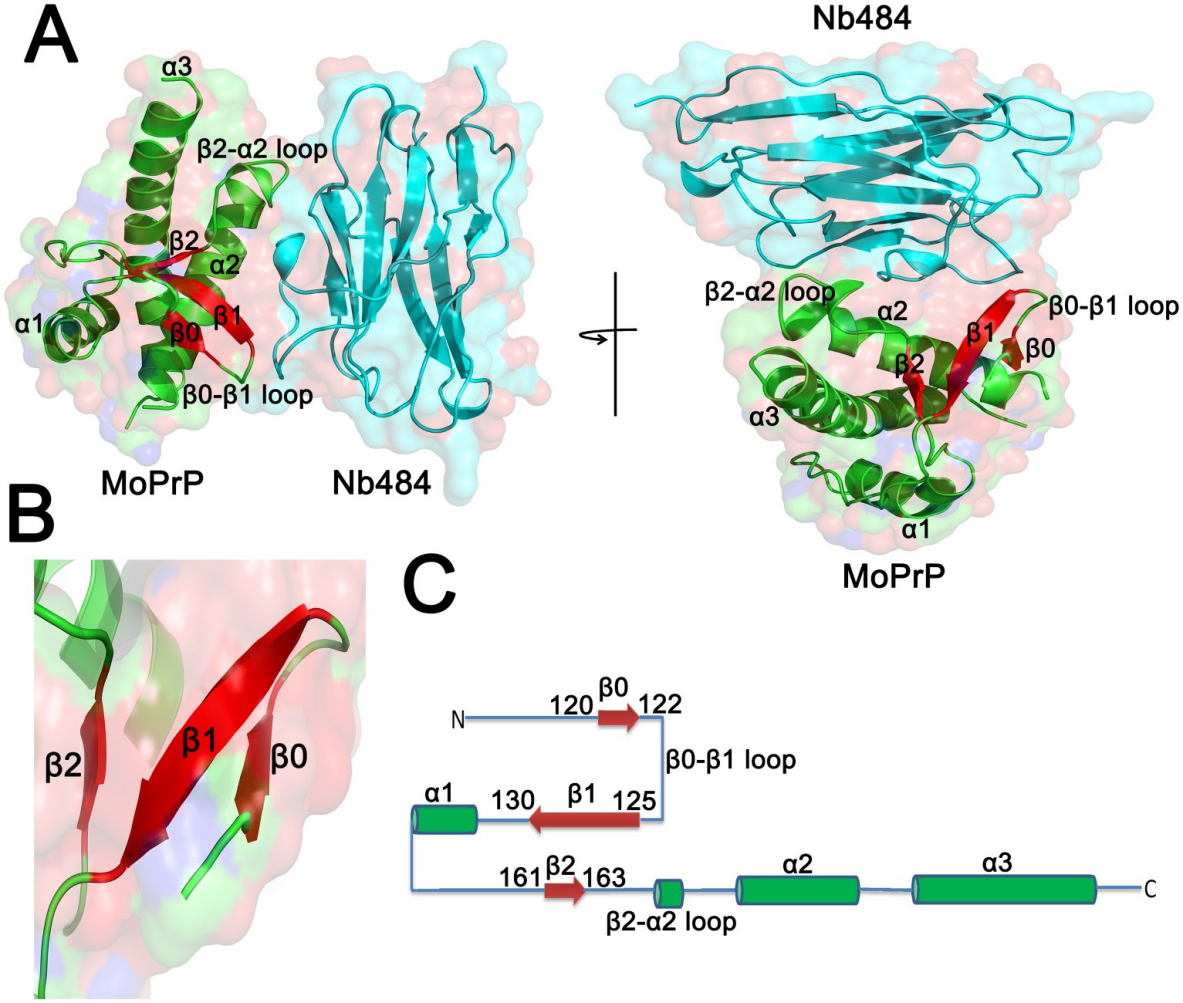
Crystal structure of the MoPrP(89–230) in complex with Nb484 at pH 8.0. (A) Ribbon representation of the MoPrP•Nb484 complex shown in two orientations, the MoPrP is shown in green and Nb484 highlighted in cyan. (B) Cartoon representation of the three-stranded antiparallel β-sheet shown in red. (C) The β0-β1-α1-β2-α2-α3 topology of MoPrP(89–230).

The overall structure of the MoPrP(89–230) in complex with Nb484 consists of three antiparallel β-strands (β0: residues 120–122, β1: residues 125–130 and β2: residues 161–163, human numbering, for the convenience of direct comparison between Mouse PrP and Human PrP) and three α-helices (α1: residues 144–153, α2: residues 172–190 and α3: residues 200–226) (Fig 1A and 1C). Similar to the human protein, the palindromic motif, AGAAAAGA, of MoPrP (residues 113–120) adopts a stable β-hairpin fold to form a three-stranded antiparallel β-sheet with the β1 and β2 strands (Fig 1B). It thus appears that the MoPrP structure also adopts a more elaborate β0-β1-α1-β2-α2-α3 fold than the canonical PrP^C^ fold[12]. MoPrP(89–230) interacts with Nb484 through a discontinuous binding epitope comprising residues 123 and 125 of the β0-β1 loop; residue 128 of β1 strand; residues 164, 167, 168, and169 of the β2-α2 loop; and residues 173, 174, 177, 178, 182, 185 and 189 of the α2-helix. A detailed overview of all the interactions occurring between the folded domain of MoPrP and Nb484 is shown in S1 Fig and S1 Table.

In both MoPrP structures, Nb484 binds the hydrophobic domain at Gly123 and Leu125 to stabilize a third β-strand (β0), which forms a three-stranded anti-parallel β-sheet with the extended strands β1 and β2. The β0 strand in both MoPrP structures (residues 120–122) is shorter than in the one reported in human structure (residues 118–122)[12] (S2 Fig).

We also crystallized Nb484 alone and solved its structure to a high resolution (1.2 Å) by X-ray crystallography. Nb484 contains three complementarity determining regions (CDRs), each noncontiguous with the others (termed CDR1, CDR2, CDR3) (S3A and S3F Fig). These regions account for the specificity of the antibody for a particular antigenic determinant. Remarkably, the structure of the free Nb484 changes in the CDR3 from the same nanobody in complex with the MoPrP (S3A and S3B Fig). More specifically, the orientation of the CDR3 changes upon binding with the MoPrP, thus interacting with β0-β1 loop and β2-α2 loop. The B-factors indicate that the CDR3 of the Nb484 is significantly more rigid in the MoPrP•Nb484 complex as compared to the nanobody alone (S3C and S3D Fig). Specifically, Ile102 and Tyr103 of the Nb484 undergo major structural rearrangement towards Gly123 and stabilize the β0-β1 loop of MoPrP. In addition, Arg106 stabilizes α2 of MoPrP by binding to Asn174, His177 and Asp178, while Ala107 stabilizes the β2-α2 loop of MoPrP by binding to Gln168 and Tyr169 (S3E Fig). It thus appears that Nb484 undergoes structural changes in the CDR3 to enable a greater complementarity between the nanobody and MoPrP (S3E and S4 Figs).

### The inhibitory effect on prion conversion by Nb484 is epitope-specific

**Fig 2:**
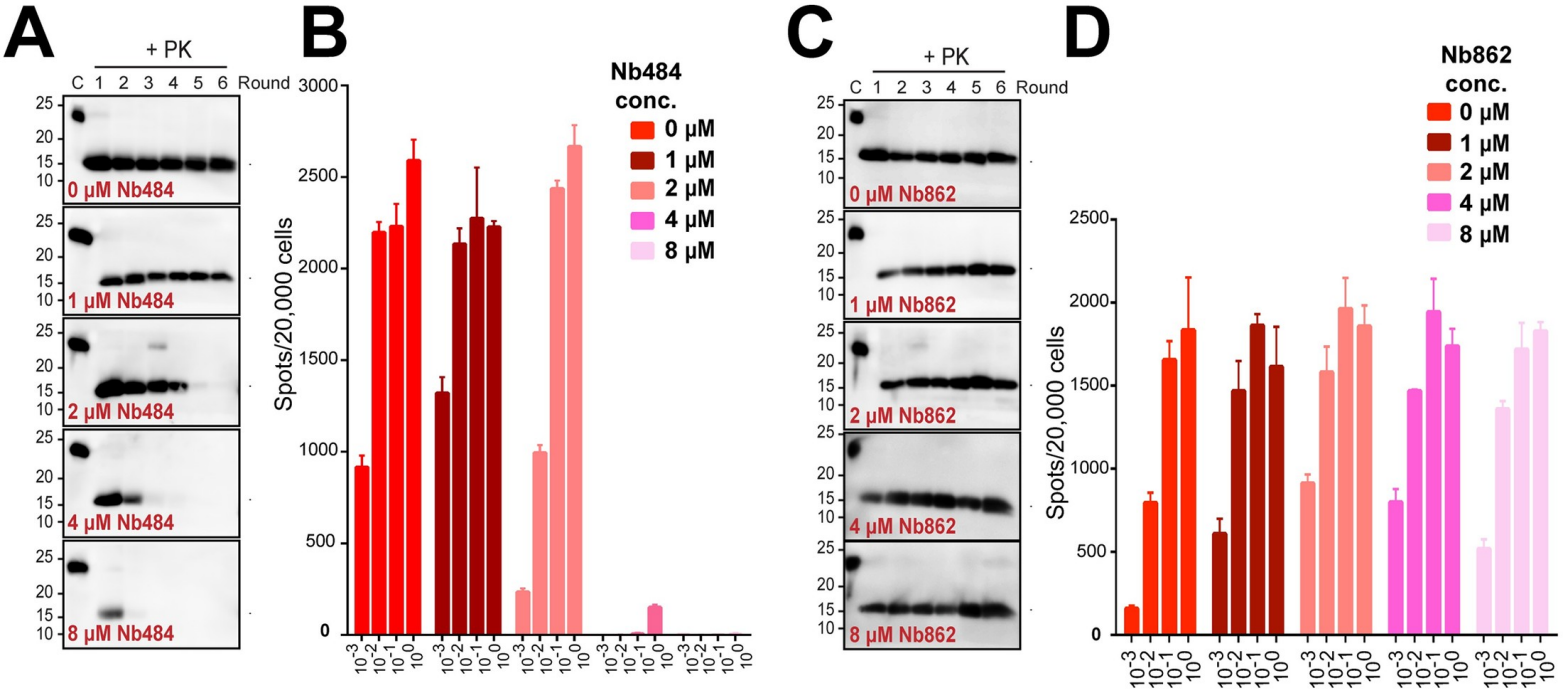
Effect of nanobodies on the prion amplification. (A) Inhibition of prion propagation by different concentrations (1, 2, 4 and 8 μM) of Nb484 in Protein Misfolding Cyclic amplification (PMCA) for six consecutive rounds. (B) Elispot cell culture assay of prion infectivity from PMCA samples (round 6) for Nb484. (C) Inhibition of prion propagation by different concentrations (1, 2, 4 and 8 μM) of Nb862 in PMCA. (D) Elispot cell culture assay of prion infectivity from PMCA samples (round 6) for Nb862. CAD5 cells were infected with serial 10-fold dilutions of round six of PMCA products.

We previously reported that Nb484 inhibits prion propagation in a scrapie-infected GT1 mouse hypothalamic (ScGT1) cell line in a dose-dependent manner and cures the scrapie-infected cells[12]. Here, we tested the ability of Nb484 to inhibit prion conversion in our recently developed recombinant prion propagation system, in which the non-infectious, α-helical recombinant mouse PrP (recPrP) is converted into the highly infectious recombinant prion (recPrP^Sc^) by PMCA reaction supplemented with two auxiliary cofactors, phospholipid POPG and RNA molecules[13]. In the PMCA reactions seeded with recPrP^Sc^, robust recombinant prion amplification was observed in the absence of Nb484 (Fig 2A), while in the presence of Nb484 (1, 2, 4 and 8 μM Nb484), the propagation of recPrP^Sc^ was blocked depending on the concentration of Nb484 (Fig 2A). To further evaluate the inhibitory effect of Nb484 on prion infectivity, we collected PMCA products from the 6^th^ round and performed the cell-based prion infectivity assay[14]. Our results revealed that Nb484 inhibits the propagation of mouse prion infectivity as well (Fig 2B). ELISA was performed to evaluate the binding of Nb484 to recPrP and recPrP^Sc^. Our result confirmed that Nb484 has a high affinity for recPrP, as previously reported (K_d_ = 40 nM)[12], but revealed that its binding to the infectious recPrP^Sc^ is minimal (S5A Fig, Nb484), indicating that Nb484 exerts its inhibitory effect on prion propagation through binding to the non-infectious recPrP (Fig 2A and 2B).

It has been suggested that the inhibitory efficacy of conventional antibodies on prion conversion positively correlates with their binding affinity for PrP^C^, *i*.*e*. higher affinity, stronger inhibition[11]. To evaluate whether such correlation exists for nanobodies, we tested the inhibitory abilities of two extra nanobodies, Nb862 and Nb486, which have higher and lower binding affinity for MoPrP, respectively, compared to Nb484 (S2 Table and **ref**.[12]). Interestingly, neither Nb862 nor Nb486 inhibits the prion propagation of MoPrP in the seeded PMCA reactions (Fig 2C and 2D and S5B Fig), suggesting Nb484 inhibits prion conversion in an epitope-specific manner.

### Nb484 inhibits recombinant prion conversion by competitively binding to the hydrophobic region of MoPrP

Structure-function studies have shown that anti-PrP monoclonal antibody ICSM18, a therapeutic antibody, blocks prion amplification through binding to and stabilizing the α-helix 1 of PrP[11]. The crystal structure of MoPrP(89–230)•Nb484 complex clearly reveals the stabilization of β0-β1 loop and β2-α2 loop (Fig 1 and S1 and S2 Figs). To investigate the underlying mechanism of the inhibitory effect of Nb484 on mouse recombinant prion propagation, we started with the lipid binding and PK-digestion assay.

**Fig 3:**
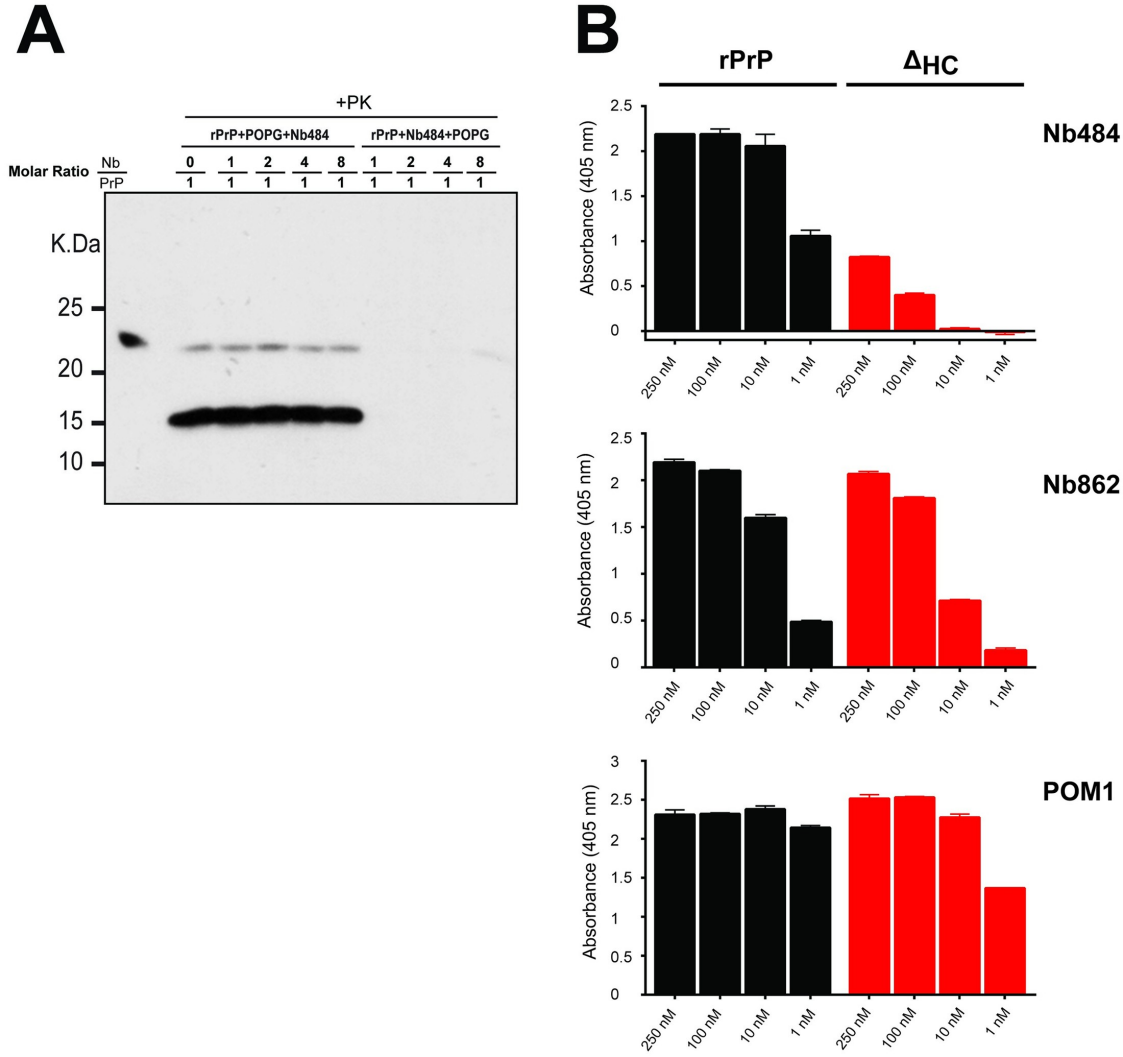
Nb484 interacting with the hydrophobic region of PrP^C^. (A) Influence of Nb484 on the interaction of PrP and POPG synthetic lipid. For rPrP+POPG+Nb484, recombinant mouse PrP was incubated with POPG before mixed with Nb484 at different molar ratios (Nb484:recPrP = 0, 1, 2, 4, or 8:1). For rPrP+Nb484+POPG, recombinant mouse PrP was incubated with Nb484 at different molar ratios (Nb484:recPrP = 0, 1, 2, 4, or 8:1) before mixed with POPG. PK-resistant PrP was detected using POM1 antibody. (B) Representative ELISA results show that both Nb484 and Nb862 bind strongly to rPrP. Nb484 has very week binding to Δ_HC_, but Nb862 shows strong binding signal to Δ_HC_. POM1 antibody was used as a primary antibody to determine the binding signal. POM1 displayed similar binding for both rPrP and Δ_HC_. All results are the average of 3 replicates.rPrP: MoPrP(23–230), Δ_HC_: MoPrPΔ_HC_.

We have previously shown that anionic phospholipid POPG, one of the cofactors that facilitate the propagation of recombinant prion, can bind to recPrP and induce conformational changes and C-terminal Proteinase K (PK)-resistance of recPrP[13]. The recPrP-POPG interaction is initiated by the electrostatic interaction between the positively charged residues of PrP and the negatively charged head groups of POPG and followed by the hydrophobic interactions between the hydrophobic region of PrP (112–135, human numbering) and the hydrophobic acyl chains of POPG, and such hydrophobic interaction is essential for the POPG-induced C-terminal PK-resistance[15, 16]. Since the novel β0 strand (120–122) and β0-β1 loop (123–125) stabilized by Nb484 locate within the MoPrP hydrophobic region (112–135), we tested if Nb484 can block the access of POPG to the hydrophobic region of MoPrP and then if it can inhibit POPG-induced recPrP prion-like conversion. Increased amounts of Nb484 were incubated with either the recPrP•POPG mixture or recPrP alone prior to the addition of POPG, and the development of the C-terminal PK-resistance was used to track the conformational change of recPrP. As previously reported[15], recPrP underwent structural changes and acquired the C-terminal PK-resistance when incubated with POPG alone (Fig 3A, 0 μM Nb484). Interestingly, the addition of Nb484 at various concentrations to the recPrP•POPG mixture had no effect on the lipid-induced C-terminal PK-resistance (Fig 3A, 7.8, 15.6, 31.2 and 62.4 μM Nb484). However, when Nb484 was allowed to interact with recPrP prior to being mixed with POPG, a dose-dependent inhibition of POPG-induced PK-resistance of recPrP was found (Fig 3A and S6 Fig), suggesting a competition between Nb484 and POPG for binding to the hydrophobic region of MoPrP.

The competitive binding of Nb484 or POPG to recPrP was further supported by results of the discontinuous iodixanol density gradient floatation assay (S7 Fig). In this assay, various mixtures composed of recPrP, Nb484 and/or POPG were loaded at the bottom fraction of the density gradient and subjected to ultracentrifugation. If the sample contains lipids and there is an interaction between lipids and other components, the lipid-bound components will migrate along with the lipids to the top fractions of the gradient due to the low density of lipids; otherwise the non-lipid components will remain at the bottom fractions. As previously reported[15, 16], recPrP alone stayed at the bottom fractions and recPrP•POPG mixture migrated to the top fractions (S7A Fig). When recPrP was first incubated with POPG and then mixed with Nb484, recPrP floated to the top fractions, but most of Nb484 was left at the bottom fractions (S7B Fig, [recPrP•POPG]+Nb484), showing recPrP-POPG interaction prohibited Nb484 binding to recPrP. Interestingly, when the recPrP•Nb484 mixture was incubated with POPG, both recPrP and most of Nb484 were found in the top fractions (S7B Fig, [recPrP•Nb484]+POPG). It is most likely that recPrP in the mixture of [recPrP•Nb484]+POPG binds to POPG through its positively charged residues instead of the hydrophobic region that is occupied by Nb484.

To evaluate if the availability of the hydrophobic region of MoPrP for conformational change is indeed involved in forming an infectious prion, we generated a recPrP mutant, in which a segment (from amino acid 112 to 132, human numbering) of the hydrophobic region was deleted (designated as MoPrPΔ_HC_). This mutant does not support recPrP^Sc^-seeded prion propagation in PMCA (S8 Fig). Relative to the full-length recPrP, binding of MoPrPΔ_HC_ to Nb484 dramatically decreases (Fig 3B, Nb484), indicating that the β0-β1 loop is the major contributor to the interactions between recPrP and Nb484. In contrast, Nb862, the nanobody that does not inhibit prion propagation, binds to both full-length recPrP and MoPrPΔ_HC_ similarly (Fig 3B, Nb862), indicating that the epitope for Nb862 does not include residues within the hydrophobic region of PrP. The epitope for a conformational anti-PrP monoclonal antibody POM1 has been determined, via a structural study, to include residues 138–147, which encompass the β1-α1 loop and part of α-helix 1 and are just outside of the hydrophobic region, and three discontinuous residues 204, 208 and 212 on α-helix 3[17, 18]. The ELISA result shows that the binding of POM1 to full-length recPrP and MoPrPΔ_HC_ are essentially the same (Fig 3B, POM1). Together with the recPrP^Sc^ propagation inhibition results, the ELISA data strongly suggests that Nb484 binds to recPrP hydrophobic region, blocks the interactions between recPrP and cofactor molecules, and inhibits PrP conversion and prion propagation.

To assess the effects of β2-α2 loop stabilization by Nb484 on recPrP conformational change and prion conversion, we generated a “rigid loop” MoPrP variant by introducing both S170N and N174T mutations (human numbering)[19] (designated as MoPrP^S170N/N174T^) and subjected the mutant MoPrP to both lipid interaction and PMCA assays. Interestingly, the stabilized β2-α2 loop does not affect the interaction between MoPrP^S170N/N174T^ and POPG (S10A Fig, molar ratio of Nb484:PrP = 0:1), and the competition binding assay reveals that MoPrP^S170N/N174T^ behaves like wild-type MoPrP (S10A Fig, in comparison with S6 Fig and Fig 3A). Furthermore, MoPrP^S170N/N174T^can be readily converted to prion in our recombinant PMCA assay (S10B Fig), indicating that stabilization of β2-α2 loop would not inhibit recombinant prion propagation. Taken together, our results support that Nb484 inhibits recombinant prion conversion by competitively binding to the hydrophobic region of MoPrP.

### Nb484 does not induce neurotoxicity in organotypic cultured slices

**Fig 4:**
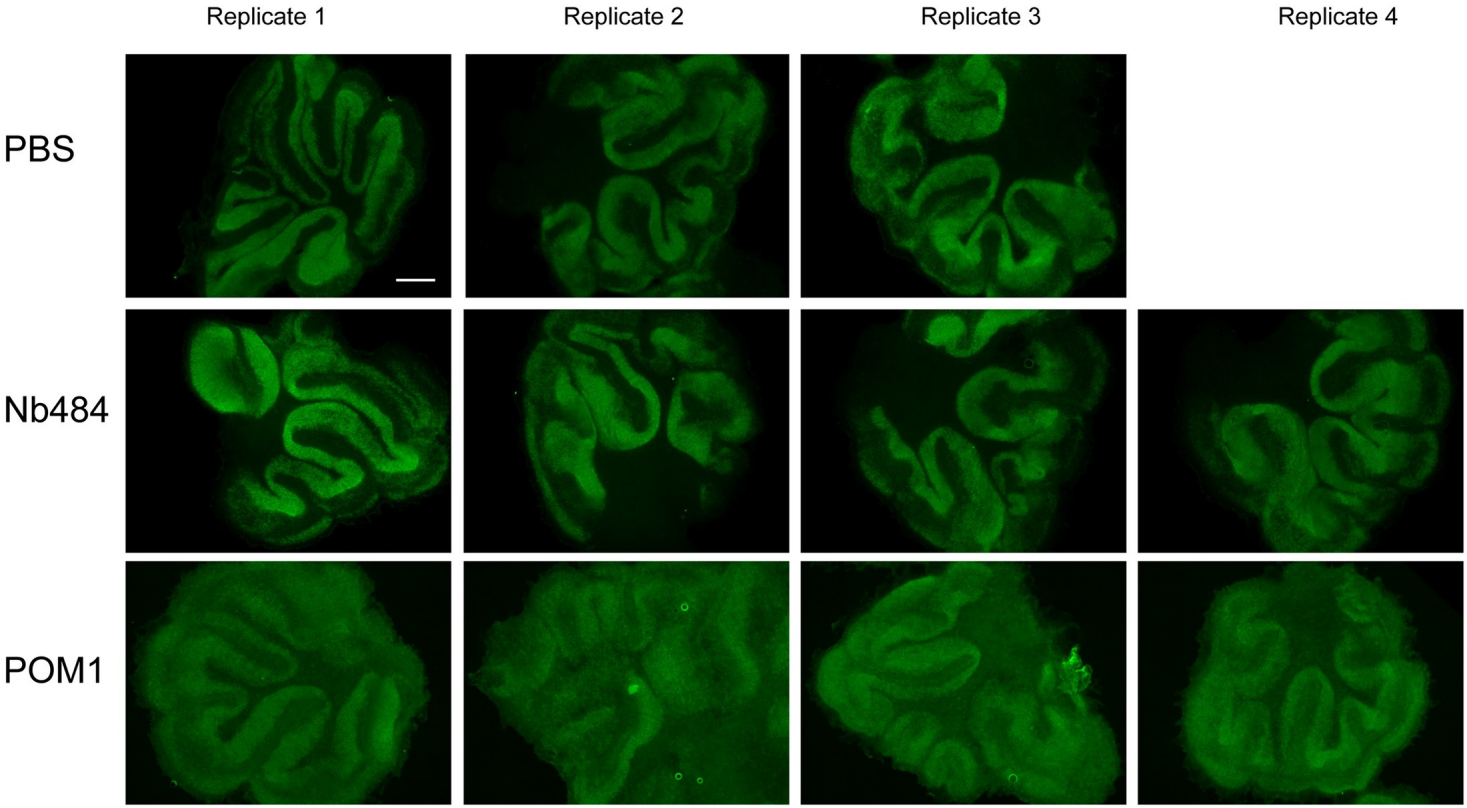
Nb484 did not induce neurotoxicity in Tga20 mice organotypic slices comparing to POM1 antibody. NeuN staining showing that POM1 has strong toxicity similar to the pervious results obtained by Aguzzi group [18]. Nb484 shows no neurotoxicity in Tga20 mice organotypic slices similar to the slices treated with PBS. Slices were stained with IgG1 antibodies to NeuN. The white bar is 100 μm.

It has been shown that certain antibodies (including POM1) that bind to the C-terminal globular domain of MoPrP (residues 124–230) induce strong neurotoxicity in mice and cerebellar organotypic cultured slices[18]. To evaluate whether Nb484, which binds discontinuous epitopes involving both the hydrophobic region and C-terminal domain of PrP, causes neurotoxicity in PrP^C^ expressing cells, we cultured cerebellar organotypic cultured slices from tga20 transgenic mice and treated slices with either POM1 or Nb484. Consistent with previous report, POM1 at 270 nM induced rapid neurotoxicity in the cultured slices in two weeks (Fig 4). In contrast, Nb484 at concentration of 270 nM or 2700 nM did not elicit any obvious neurotoxicity (Fig 4 and S11 Fig), revealing that Nb484 binding to the hydrophobic region does not cause neurotoxicity and confirming that the neurotoxicity induced by globular domain-ligand binding- is mediated through specific regions of PrP[18].

## Discussion

Despite great advances in understanding prion propagation, the molecular mechanism underlying the conformational conversion from normal PrP^C^ to the infectious PrP^Sc^ remains largely unknown. We previously reported that Nb484, an anti-PrP nanobody, could inhibit such PrP^C^-to-PrP^Sc^ conversion in chronically prion infected mouse hypothalamic cells (scrapie-infected GT1 mouse hypothalamic (ScGT1) cell line)[12]. To better understand how Nb484 recognizes MoPrP and inhibits prion propagation *in vitro*, crystal structures of the MoPrP(89–230)•Nb484 complexes and Nb484 alone were determined in current study. The discontinuous epitope of MoPrP that interacts with Nb484 includes residues 123–125 in the β0- β1 loop, residues 164–170 in the β2–α2 loop and residues 174–185 in the α2-helix.

The structure of MoPrP•Nb484 complex confirms structural insights into the folding of the hydrophobic region from residues 118 to 135. Similar to the HuPrP•Nb484 complex, the C-terminus of the palindromic segment participates in the formation of an additional β-strand (residues 120–122, designated as β0), which packs with β1 and β2 into three anti-parallel β-sheets. Remarkably, Nb484 binds to Y128 between G127 and M129, which leads to an extended β1 strand (residues 125–130). The β0 and β1 strands are connected with a defined loop, termed β0-β1 loop (Fig 1), which forms a 2:2 IP type β-hairpin between β0 and β1 strands (S9 Fig). In this loop, Gly123 and Gly124 occupy a type I′ β-turn and are stabilized by H-bonds located between Val122 and Leu125. In addition, the formation of this β-hairpin exposes several backbone hydrogen donors and acceptors to solvent (S4 Fig). These amino acids are prone to stacking with other β-strands in parallel or anti-parallel fashion to build β-sheeted amyloid fibers (S4 Fig). Our findings suggest that the binding of Nb484 to this region not only stabilizes the PrP hydrophobic region, but also prevents the amyloid fiber formation.

The MoPrP-Nb484 interactions also significantly stabilize the β2–α2 loop at the C-terminal domain, leading to a rigid β2–α2 loop (S3G Fig). The rigidity of this loop was initially linked to the efficiency of the interspecies prion transmission, that is, PrP of a species with a rigid β2–α2 loop will be susceptible to infection by prions from another species with a similar rigid β2–α2 loop[19]. However, in an elegant follow-up transmission study, it has been shown that the efficiency of prion conversion correlates with primary sequence homology rather than the rigidity of β2–α2 loop in different PrP species[20]. In our hands, the stabilized β2–α2 loop, resulting from amino acid substitutions, namely S170N and N174T, does not affect the binding between the MoPrP and POPG (S10A Fig), which is not surprising since we have shown previously that POPG binds to the positively charged residues and the hydrophobic region of MoPrP, but not the folded C-terminus[16]. Interestingly, the rigid β2–α2 loop in MoPrP^S170N/N174T^ does not prevent its conversion to recombinant prion (S10B Fig). Therefore, it is unlikely that the β2–α2 loop, stabilized by Nb484, accounts for the inhibitory effect of Nb484 on prion conversion.

On the other hand, the β0 strand (120–122) and β0-β1 loop (123–125), stabilized by Nb484, locate within the PrP hydrophobic region (112–135), which is the most conserved motif of PrP among all species[21]. PrP-null mice expressing PrP_Δ94–134_ or PrP_Δ105–125_, both of which lack the hydrophobic region, spontaneously developed rapid and lethal neurodegenerative illnesses[22, 23]. Although such neurodegenerations are distinct from those observed in prion infected mice and can be rescued by co-expression of wild-type PrP^C^, these observations suggest that the alteration of the hydrophobic region may affect its binding to a ligand and leads to neurotoxicity[22, 23]. The hydrophobic region also harbors multiple mutation sites that are associated with inherited human prion disease, such as G114V and A117V mutations in the palindromic sequence AGAAAAGA (113–120), which cause early onset Gerstmann-Straussler-Scheinker disease (GSS)[24, 25]. Removing this palindromic sequence in PrP led to failed PrP^Sc^ conversion in prion-infected ScN2a cells and altered PrP aggregation in yeast, suggesting that the AGAAAAGA sequence is not only required for forming a prion but also likely involves in the PrP^Sc^-PrP^C^ interaction to initiate the conformational conversion[26]. It has been shown that the hydrophobicity of the palindromic region is critical for the neurotoxicity and fibrillogenicity of the PrP106-126 peptide[27, 28]. Low-resolution spectroscopy data also indicate that the hydrophobic AGAAAAGA motif may adopt multiple discrete conformations, suggesting this region is metastable, and depending on intermolecular interactions, forms various structures[29, 30]. Consistent with this idea, the Eisenberg and Yee groups have identified that multiple segments in the hydrophobic region could form different steric zipper structures, including ^113^AGAAAA^118^, ^119^GAVVGG^124^, ^126^GGYMLG^131^, ^127^GYMLGS^132^, ^126^GGYVLG^131^ and ^127^GYVLGS^132^ with class 7, 4, 7, 8, 8 and 8, respectively[31–33].

The therapeutic anti-PrP monoclonal antibody ICSM18 inhibits prion conversion by binding strongly to the whole α-helix 1 and the binding affinity of an antibody for PrP has been suggested to correlate with its ability to inhibit PrP^Sc^ propagation[11]. Interestingly, we found that Nb862, which has a much higher binding affinity for MoPrP (0.158 nM vs. 40 nM of Nb484), does not affect recPrP^Sc^ propagation at all (Fig 2C and 2D), suggesting that, for a nanobody that is much smaller compared to the conventional antibody, the binding epitope is more critical for its inhibitory effect. Among the different epitopes of Nb484, the hydrophobic region plays the most critical role in forming an infectious prion. Using the recombinant prion propagation system with defined components, Nb484 inhibits recPrP^Sc^ propagation in a dose-dependent fashion (Fig 2A and 2B). We have also found that the hydrophobic region is required for an effective binding of Nb484 to PrP (Fig 3B) and Nb484 blocks the hydrophobic interactions between PrP and POPG (Fig 3A and S6 and S7 Figs). These data demonstrate that Nb484 inhibits prion conversion through a competitive inhibitory mechanism.

The conversion of PrP^C^ to PrP^Sc^ involves major conformational rearrangements[8, 34]. However, it is unlikely that the whole C-terminal region of PrP^C^ undergoes dramatic conformational changes simultaneously, and the conversion is likely initiated at one or a few parts of PrP^C^ in contact with PrP^Sc^ directly or through certain ligands[4]. The hydrophobic region has been suggested to be involved in the PrP^C^-PrP^Sc^ interaction during prion conversion[21]. Moreover, an anti-prion phenothiazine compound has been shown to bind to a “hydrophobic pocket” that encompasses part of the hydrophobic region and stabilize the PrP molecule[35]. Our previous study found that the hydrophobic region is indispensable to the formation of POPG-induced C-terminal PK-resistance[16]. Our current data reveal that the hydrophobic region is also critical for the inhibitory nanobody Nb484 to bind PrP (Fig 3B). Therefore, it is reasonable to speculate that the hydrophobic region represents one of the misfolding initiation sites for the prion conversion.

**Fig 5:**
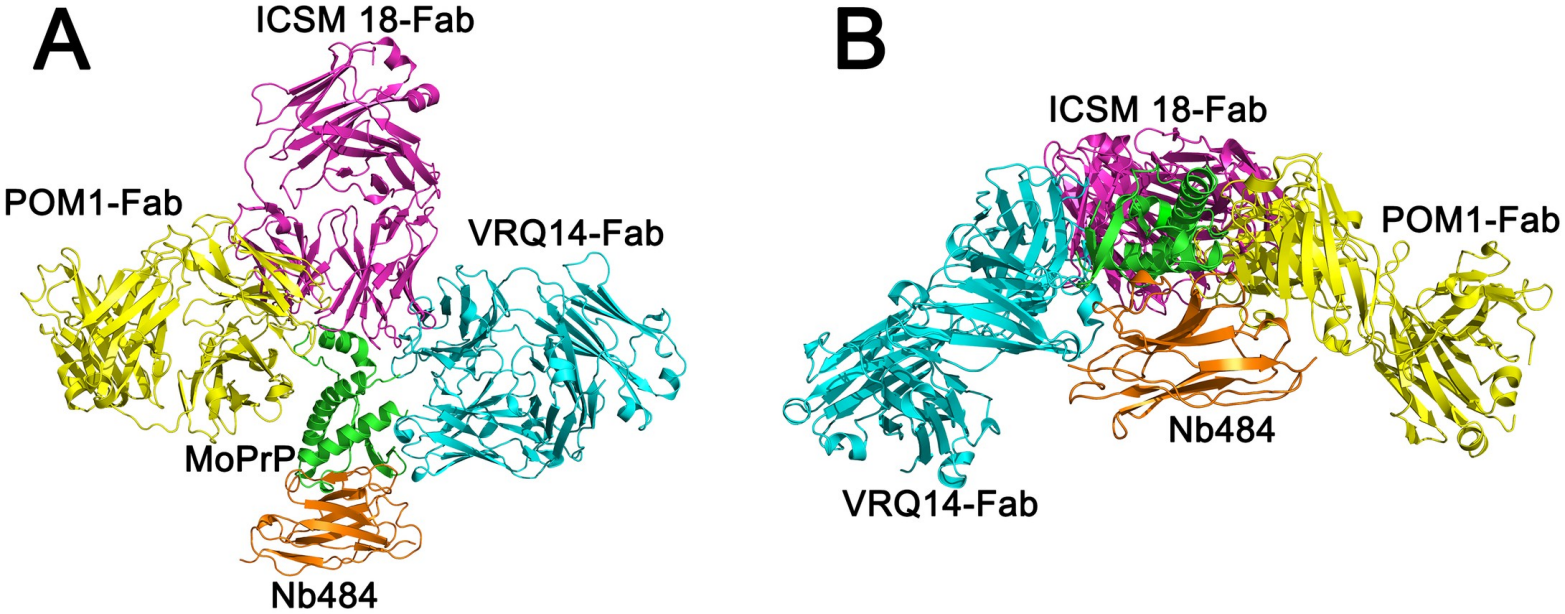
Superimposition of different MoPrP, HuPrP and ovine PrP structures in complex with Fab and Nb484. (A) Front view, all prion proteins are shown in green, heavy and light chains of POM1 Fab (PDB 4H88) are shown in yellow, ICSM18 (PDB 2W9E) in pink, VRQ14 Fab (PDB 1TPX) in cyan and Nb484 in brown. (B) Top view of the superposition.

Many anti-prion compounds have been identified to inhibit prion replications in cell cultures and cell free conversion assays, such as polyanions[36], iododoxorubicin[37], tetracycline[37], Congo Red[38], polyene antibiotics[39] and quinacrine[39]. However, these compounds have limited applications in treating human prion diseases mainly due to their limited ability to cross the blood brain barrier (BBB)[40, 41]. It has been reported that an anti-prion nanobody is able to cross BBB *in vitro* and *in vivo*, making them particularly interesting for therapeutic application[40]. As mentioned above, passive immunotherapy with anti-PrP antibodies is a promising therapeutic approach against prion disease, but the observations that some anti-PrP antibodies induce rapid neurotoxicity cause some concerns about this strategy[18, 42]. Interestingly, the inhibitory Nb484 does not elicit any toxicity in cultured cerebellar organotypic slices (Fig 4 and S11 Fig). Structural superimposition of MoPrP bound to Nb484 with MoPrP•POM1 Fab[35], huPrP•ICSM18 Fab[17] and ovPrP•VRQ14 Fab[43] clearly reveals the distinct binding orientations of each antibody (Fig 5 and S3 Table), which may be implicated in the antibody-induced neurotoxicity and warrants further investigations.

In summary, we have determined the structure of the MoPrP•Nb484 complexes, providing new structure-function evidence to support a critical role of the hydrophobic region of PrP in the formation of an infectious prion. Moreover, our data indicate an epitope-dependent inhibitory or neurotoxic mechanism for each individual antibody/nanobody. With this work we have identified a PrP^C^ region likely to be responsible for prion conversion and discovered a new potential therapeutic nanobody for prion diseases.

## Materials and methods

### Cloning, expression and purification of recombinant MoPrP(89–230)

Cloning of MoPrP(89–230) into pET-28a (Novagen) was performed as described previously[44]. Protein expression and purification was achieved according to our protocol[45]. In this method, MoPrP(89–230) was co-expressed with QSOX in *E*. *coli* Rosetta (DE3) pLysS. 10 mL of pre-culture supplemented with the appreciate antibiotics (100 μg/mL ampicillin and 25 μg/mL kanamycin) and were used to inoculate 1L of L.B medium. Cells were growing at 37 °C and induced at A600 = 0.7 by adding 1 mM isopropyl-b-D-thiogalactopyranoside (IPTG) and temperature was shifted to 15 °C for 16 h. Cells were collected by centrifugation (15 min at 15,000g). Next, pellets were re-suspended to a density of 0.1 g of cell paste/mL in lysing buffer (50 mM potassium phosphate, pH 7.5, 300 mM NaCl supplemented with 0.1 mg/mL lysozyme, 0.1 mg/mL AEBSF and 1 μg/mL leupeptin). Cells were lysed using a French press (10,000 psi) and followed by centrifugation at 4 °C for 60 min at 20,000 rpm. The collected supernatant was loaded on a 5 mL Histrap Ni-NTA column (GE-healthcare) previously equilibrated with equilibration buffer (50 mM potassium phosphate pH 7.5, 300 mM NaCl, 10 mM imidazole). The column was washed with five column volumes (CV) of washing buffer: 50 mM potassium phosphate pH 7.5, 1 M NaCl, 50 mM imidazole, followed by ten CV of 50 mM potassium phosphate pH 6.0, 1 M NaCl, 50 mM imidazole. The protein was eluted with a gradient of imidazole from 50 mM to 500 mM in 50 mM potassium phosphate pH 7.5. The eluted MoPrP(89–230) fractions were loaded on a SDS/PAGE to evaluate purity, then pooled, and concentrated for a second purification step. The concentrated pool was applied onto a Superdex75 HR 10/30 column (GE Healthcare) and eluted with 20 mM Tris-HCl pH 7.5 containing 150 mM NaCl. The elution peak was again loaded on SDS/PAGE. The fraction containing only MoPrP(89–230) was collected for a dialysis against 10 mM sodium acetate pH 4.6, 1 mM EDTA followed by the final dialysis buffer of 10 mM sodium acetate pH 4.6. Protein aliquots were stored at -80 °C until further usage.

### Expression and purification of recPrP23-230 and MoPrPΔ_HC_

Full-length recPrP23-230 was expressed in *E*. *coli* BL21 (DE3) and purified according to the previous reported protocols[46, 47]. MoPrP**Δ**_HC_ is a mutant with deletion of the hydrophobic domain (amino acids 112–132 deletion, human numbering). MoPrPΔ_HC_ was constructed, expressed and purified according to the previous reported protocol[16].

### Construction, expression and purification of MoPrP^S170N/N174T^

Mutations S170N and N174T in MoPrP were generated using the QuikChange site-directed mutagenesis kit (Stratagene) from pPROEX-HT-b vector containing mouse PrP23–230. The mutant was expressed in *E*. *coli* BL21 (DE3) and the purification was carried out as previously described[16].

### Generation, expression and purification of nanobodies

Nb484 and Nb486 were generated from llama immunized 6 times bi-weekly with 200 μg of purified recombinant MoPrP(23–230). Nb484 and Nb486 were generated and selected according to our protocol[48]. Nb862 were generated by immunizing llama 6 times bi-weekly with approximately 200 μg of PrP^Sc^ chemically purified from ScGT1 cell[12]. Nb484, Nb486 and Nb862 were cloned into (pHEN6) vector bearing a C-terminal His6 tag and a pelB signal peptide for periplasmic protein expression. The plasmid was transformed into *E*. *coli* WK6 cells and a single colony was grown overnight in LB medium 100 μg ml^−1^ ampicillin. Next, 10 ml of pre-culture was used to inoculate 1 L TB medium containing 100 μg ml^−1^ ampicillin, 2 mM MgCl_2_ and 0.1% glucose and grown overnight at 37 °C. Cultures were grown to A600 = 0.7 and induced with 1mM IPTG followed by shifting the growing temperature to 28 °C overnight. Cells were harvested by centrifugation and lysed in ice-cold buffer (50 mM Tris pH 8.0, 12.5 mM EDTA and 0.125 M sucrose), then centrifuged to remove cell debris. The periplasmic extract of the nanobody was applied to Ni-NTA column and washed with buffer: 50 mM Na_2_HPO_4_, 1 M NaCl pH 7 followed by 50 mM NaH_2_PO_4_, 1 M NaCl pH 6.0. The purified nanobody with eluted using 10 ml of 50 mM sodium acetate pH 4.7, 1 M NaCl and neutralized with 2 ml 1 M Tris–HCl pH 7.5. Next, the collected fraction was concentrated and applied for another purification step using gel filtration on a Superdex 75 HR 10/30 column equilibrated in 20 mM Tris–HCl pH 7.5, 150 mM NaCl. Fractions were collected, concentrated and applied for SDS PAGE and showed 99% pure nanobody.

### Purification and crystallization of MoPrP(89–230)•Nb484 complex

MoPrP(89–230) and Nb484 were mixed in an equimolar ratio in order to form the protein complex. Presence of a stable complex was monitored by analytical SEC using a Superdex 75 HR 10/30 column (GE Healthcare Life Sciences) in a buffer running containing 20 mM Tris-HCl pH 7.5, 150 mM NaCl. Two crystals of MoPrP(89–230)•Nb484 complexes were grown after using several commercial screening conditions (MD-proplex, Index, Crystal Screen, Crystal Screen 2, PACT, JCSG, JBScreen Classic 1–4 HTS, JBScreen Classic 5–8 HTS and JBScreen Basic HTS) in 96-well Intelli-plates (Hampton research). The first complex crystal was grown at 20 mg/mL complex in MD-proplex screen in B10 (0.15 M ammonium sulfate, 0.1 M MES pH6.0, 15% w/v PEG 4000) within 2 weeks. The second complex crystal was grown at concentration 68 mg/mL complex in MD-proplex screen in G1 (0.1 M Tris pH 8, 1.5 M ammonium sulfate) within one year. Nb484 has crystallized and diffracted to 1.2 Å resolution as described previously[49]. All crystals were grown at 20 °C and cryoprotected using 15% glycerol.

### Data collection and structure determination

The MoPrP(89–230)•Nb484 complex crystal diffracted to 2.1 Å resolution and a complete dataset was collected at X06DA beamline at SLS, Paul Scherer institute, Switzerland as described previously[44]. The second crystal diffracted to 1.2 Å resolution and a complete dataset was collected at X06DA beamline at SLS, Paul Scherer institute. Both Mouse PrP structures data were processed using XDS[50]. The structures of the different MoPrP•Nb484 complexes were determined by molecular replacement (PHASER)[51] using the HuPrP•Nb484 crystal structure (PDB entry 4KML)[12]. Nb484 was processed using *iMOSFLM* as described previously[49]. The Nb484 structure was solved by molecular replacement using PDB entry 1OL0 as search model.

The data collection details and refinement statics are shown in Table 1. Models were built manually using the Crystallographic Object-Oriented Toolkit (Coot)[52] and multiple refinement rounds were performed using Refmac5[53] and Phenix[54]. Structural analyses were performed using Ligplot[55], Promotif[56], and Pisa[57].

### Protein misfolding cyclic amplification (PMCA)

To prepare substrate for propagation of recPrP^Sc^, 38.5 μL of soluble recPrP (0.75 mg/mL in deionized H_2_O) was mixed thoroughly with 81.5 μL of RNase free water (Invitrogen^™^) in a 1.5-ml siliconized microcentrifuge tube (Midsci, St. Louis). In the presence of Nb484 or Nb862, serial concentrations of (1, 2, 4 and 8 μM) or Nb462 (4 and 8 μM) was mixed with recPrP and incubated for 10 min at RT. Then, 10.7 μL of POPG (2.5 mg/mL in 20 mM Tris-HCl, pH 7.4) were added to each tube and mixtures were incubated at room temperature for 10 minutes. During the incubation, 848.36 μL of deionized H_2_O, 66.64 μL of 5% Triton X-100 and 120 μL of 10 x TN buffer (1.5 M NaCl, 100 mM Tris-HCl, pH 7.5) was added into another 1.5-ml siliconized microcentrifuge tube and mix thoroughly. The recPrP-POPG mixture was transferred to the 1.5-mL tube containing the buffer and mixed thoroughly. The mixture was incubated at room temperature for 5 minutes. Then, 45 μL of liver RNA (10 mg/mL in Nuclease Free Water) was added and the substrate mixture was thoroughly mixed, aliquoted (90 μL per tube), and stored at -80°C. All PMCA experiments were repeated twice.

### The enzyme-linked immunospot (Elispot) cell infection assay

The Elispot cell infection assay was performed according to previous studies[58] with minor modifications. Briefly, 200 μL of PMCA products at round 6 were collected and centrifuged at 100,000 × g, 4 °C for 1 hour and the pellets were washed twice with 200 μL PBS, followed by centrifugation at 100,000 × g, 4 °C for 1 hour after each wash. After the final wash, the pellets were resuspended in 200 μL of CAD5 growth media (OPTI-MEM, 5% BGS and 1% penicillin and streptomycin) and sonicated for 30 seconds with 50% output (Misonic Sonicator XL2020). Each sample was serially diluted 10, 100, and 1,000 times and 60 μL of undiluted and diluted samples were used to infect CAD5 cells. After two 1:10 splits, 20,000 CAD5 cells/well were transferred to the Millipore 96-well Elispot plates (MSIPN4W) and subjected to the Elispot assay (42). The images were taken by S6 Micro Analyzer (CTL Analyzers, LLC) and processed by the ImmunoSpot software (CTL Analyzers, LLC). The graph was generated using GraphPad Prism (GraphPad Software, Inc.).

### Enzyme-linked immunosorbent assay (ELISA)

Maxisorp 96-well plates (Nunc) were coated overnight at 4 °C with purified 2 μg/mL of Nb484 in sodium bicarbonate buffer pH 8.2. Residual protein binding sites in the wells were blocked with 2% milk in PBS for two hours at room temperature. 50 ng of recPrP23-230, PrP^Sc^ (-PK) and PrP^Sc^ (+PK) were incubated for 2 hours with the nanobody at room temperature. Then plate was incubated with 1:2500 dilution of POM1 anti-PrP antibody[59] for 2 hours. Wells were then incubated with 1:2000 dilution of goat anti-mouse HRP (Bio-Rad) for 1 hour. Absorption at 405 nm was measured 30 min after adding 100 μL of ultra TMB-ELISA (Thermo-Scientific, Product no. 34028).

### Surface Plasmon Resonance (SPR)

MoPrP(23–230) was immobilized on a CM5 chip (GE Healthcare) with Surface Plasmon Resonance (SPR) on a Biacore 3000 according to our previous reported protocol[12]. Nb862 and Nb486 used as analytes and all binding isothermes were fitted to 1 to 1 Langmuir binding model.

### Binding of Nb484 or Nb862 to MoPrP(23–230) and MoPrPΔ_HC_

Maxisorp 96-well ELISA plates were coated overnight at 4 °C with purified 2 μg/mL of Nb484 or Nb862 in sodium bicarbonate buffer pH 8.2. Residual protein binding site was blocked as above mentions. Serial concentrations (250 nM, 100 nM, 10 nM and 1 nM) of MoPrP(23–230) or MoPrPΔ_HC_ incubated 2 hours with nanobodies. POM1 anti-PrP antibody was used as a primary antibody followed by goat anti-mouse HRP as secondary antibody as previous descried. In the same time a control experiment was performed using Serial concentrations (250 nM, 100 nM, 10 nM and 1 nM) of MoPrP(23–230) or MoPrPΔ_HC_ coated in 96-well ELISA plates. The control experiment was probed by POM1 anti-PrP antibody followed by secondary antibody as described early. ELISA signal was recorded by measuring the absorption at 405 nm after adding TMB-ELISA substrate.

### PrP lipid interaction and PK digestion

15 μL recPrP (7.8 μM) was incubated for 1 hour with 10 μL of POPG (2.5 mg/mL) and 1.2 μL NaCL (5M) with the final volume of 40 μL, either before or after mixing with Nb484 in different concentrations (7.8, 15.6, 31.2 and 62.4 μM) for 10 minutes.

The PK digestions were performed by incubating 10 μL of samples with 10 μL PK (100 μg/mL) at 37 °C for 30 minutes. The reaction was stopped by adding 5 mM phenylmethyl-sulfonylfluoride (PMSF) and kept on ice for 5 minutes. The PK digested samples were separated by SDS-PAGE and the PrP was detected by immunoblot analyses with the POM1 anti-PrP antibody[59].

### Gradient floatation assay

In order to perform the discontinuous iodixanol density gradient floatation assay, we incubated recPrP or recPrP plus Nb484 with POPG for 10 minutes and the high-density phase of the iodixanol gradient was applied as previously described[15, 16]. Twelve fractions (200 μL/fraction) were collected from top to bottom of the gradient as indicated.

### Organotypic cultured slices

Cerebellar organotypic cultured slices were prepared using a vibratome from 12-day-old mice according to previously reported protocols[18, 60, 61]. Tga20 mice cerebellar organotypic cultured slices were exposed for two weeks to 270 nM and 2.7 μM of anti-PrP monoclonal antibody POM1[18] and Nb484, respectively. A control experiment was performed by exposing cerebellar organotypic cultured slices to PBS buffer.

### Accession numbers

The structures have deposited in the protein data bank as

Nb484: 6HEQ

PrP•Nb484 (at 1.2 Å resolution): 6HER

PrP•Nb484 (at 2.1 Å resolution): 6HHD

## Supporting information

S1 Fig Intermolecular contacts at the MoPrP•Nb484 interface. (A) Front-view, (B) top-view, and (C) back-view representations of the interacting residues of MoPrP (green) and Nb484 (cyan). (D) The hydrophobic interaction between MoPrP and Nb484 using Ligplot.
(TIF)

S2 Fig Comparison of MoPrP and HuPrP protein. (A) The amino-acid sequence alignment of representative PrPs showing different in the structural elements between MoPrP and HuPrP. (B) Structural comparisons of MoPrP(89–230) (MoPrP is depicted in green) with the HuPrP23-231 (PDB 4KML, X-ray) in red and MoPrP(124–230) (PDB 4H88, X-ray) in yellow.
(TIF)

S3 Fig Binding of MoPrP to Nb484 leads to structural changes in the nanobody CDR3. (A) Cartoon representation of X-rays structure of Nb484 alone showing its CDRs. The CDR1 region is shown in red, the CDR2 region in blue and CDR3 in pink. (B) Structural comparison of unbound Nb484 (cyan) with the same nanobody bound to MoPrP(89–230) in green. (C) Structural flexibility of the CDR3 in Nb484 alone and (D) the MoPrP•Nb484 complex illustrating the thermal parameter distributions in the CDR3 using the B-factor putty tube representation as implemented in PyMol. (E) The conformational changes of the interacting residues of the Nb484 with MoPrP. (F) The amino acid sequence of Nb484 with CDRs according to IMGT indicated in color. (G) Structural flexibility of the β2-α2 loop in X-ray structures. Illustration of the thermal parameter distribution in the β2-α2 loops of the MoPrP(89–230)•Nb484 complex (this study), HuPrP(23–231) alone (PDB 3HAK) and Ovine PrP(114–234) (PDB 1TPX) using the B-factor putty tube representation as implemented in PyMol.
(TIF)

S4 Fig Structure of the β0-β1 hairpin in MoPrP structure. The backbone donor and acceptor sites exposed to solvent are indicated by arrows. (A) Solvent exposed face of the β0-β1 hairpin. (B) Solvent protected face of the hairpin. (C) Model of conformation changes from cellular PrP^C^ to infectious PrP^Sc^, PrP^C^ converted to β-sheet form then followed by self-assembling into amyloid fiber by un-known mechanism. The formation β0-β1 hairpin shows backbone H-bond donor and acceptor sites to solvent. These sites can serve as a structural nucleus for the growth of amyloid fibrils, which can be inhibited by binding of Nb484.
(TIF)

S5 Fig Nb484 or Nb862 binds PrP^C^ but not PrP^Sc^. (A) ELISA assay of Nb484 or Nb862 against recombinant PrP^C^ (23–230) and PrP^Sc^ (+PK). The assay was monitored by measuring the absorbance at 405 nm. (B) Effect of Nb486 on the prion amplification. Inhibition of prion propagation by different concentrations (4 and 8 μM) of Nb486 in Protein Misfolding Cyclic amplification (PMCA) for six consecutive rounds. Nb486 has low binding affinity to recPrP, show no effect on the prion propagation in PMCA (S3 Table).
(TIF)

S6 Fig Effect of Nb484 on the interaction of PrP and POPG synthetic lipid. rPrP was incubated with Nb484 at different molar ratios (Nb484:rPrP = 0, 1, 0.5, 0.2 or 0.1:1) before mixed with POPG. POPG-induced PK-resistance was completely inhibited at Nb484:rPrP = 1:1. rPrP: MoPrP(23–230).
(TIF)

S7 Fig Effect of Nb484 on the interaction of rPrP with anionic lipid. (A) Iodixanol density gradient analysis of rPrP, rPrP + POPG, rPrP + POPG + Nb484 and rPrP + Nb484 + POPG using POM1 antibody. (B) Iodixanol density gradient analysis of rPrP + POPG + Nb484 and rPrP + Nb484 + POPG using Anti-histidine antibody to detect Nb484. rPrP: MoPrP(23–230).
(TIF)

S8 Fig The propagation of MoPrPΔ_HC_ in PMCA. (A) MoPrPΔ_HC_ was used as the substrate and seeded by rec-prion seeds in PMCA for six consecutive rounds. Four replicates of MoPrPΔ_HC_ PMCA were performed (B) Full-length mouse PrP (WT MoPrP) was used as a positive control for rec-prionPMCA (four replicates).
(TIF)

S9 Fig Structural analysis of the β0-β1 hairpin according to Promotif. (A) Schematic representation of the β0-β1 β-hairpin. Residues of the antiparallel β-strands are indicated in blue. Hydrogen bonds are represented by pink arrows. (B) Statistics of the 2:2 IP type β0-β1 β-hairpin. (C) Statistics of the β-turn.
(TIF)

S10 Fig Effect of Nb484 on the interaction of MoPrP^S170N/N174T^ with POPG. (A) MoPrP^S170N/N174T^ was incubated Nb484 at different molar ratios before mixed with POPG. PK-resistant MoPrP^S170N/N174T^ was detected using POM1 antibody. (B) Full-length WT MoPrP and MoPrP^S170N/N174T^ mutant were used as the substrates and seeded by rec-prion seeds in PMCA for three consecutive rounds. Two replicates for each substrate.
(TIF)

S11 Fig Nb484 is not toxic to organotypic slices at higher concentration. Cerebellar organotypic slices are healthy when incubated with Nb484 at concentration of 2700 nM, similar to PBS treated slices. The white bar is 100 μm.
(TIF)

S1 Table Intermolecular interactions between MoPrP(89–230) and Nb484 in the crystal structure of the complex. (DOCX)

S2 Table Binding kinetics of Nb862 and Nb486 for MoPrP(23–230). Nb862 was generated from Llamas immunized with MoPrP^Sc^ and screened against MoPrP (23–230). Nb486 was generated and screened from Llamas immunized with recombinant MoPrP(23–230).
(DOCX)

S3 Table Comparison of backbone superposition root-mean-square deviation values (RMSD, Å) of multiple PrP^C^-antibody complexes. (DOCX)
