# Supplementary figures and images for: Structural evidence for the critical role of the prion protein hydrophobic region in forming an infectious prion
Source: PLoS Pathog. 2019 Dec 9;15(12):e1008139. doi: 10.1371/journal.ppat.1008139 (PMC6922452; doi:10.1371/journal.ppat.1008139)

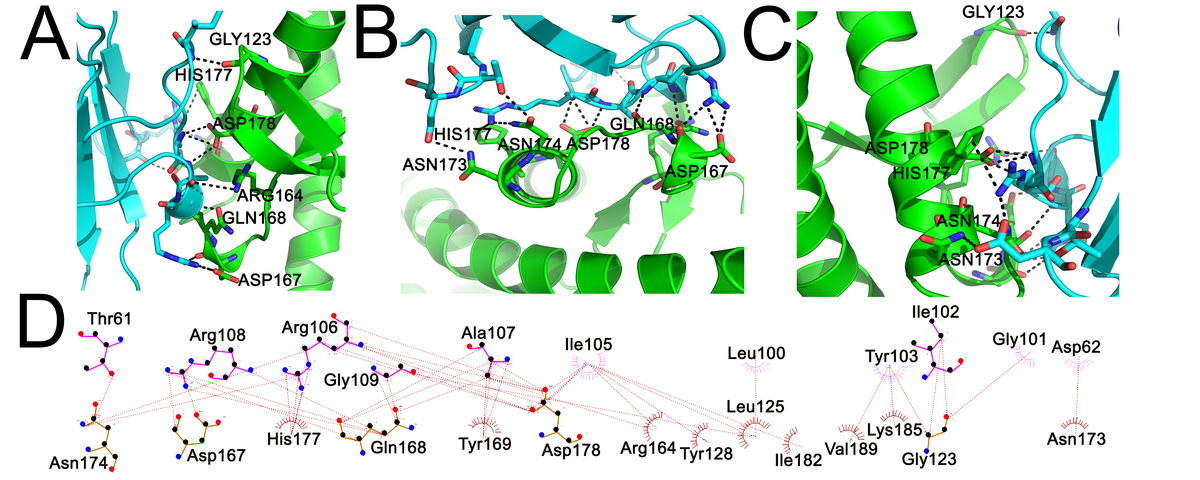

Supplement: S1 Fig — (A) Front-view, (B) top-view, and (C) back-view representations of the interacting residues of MoPrP (green) and Nb484 (cyan). (D) The hydrophobic interaction between MoPrP and Nb484 using Ligplot. (TIF) [file ppat.1008139.s001.tif]

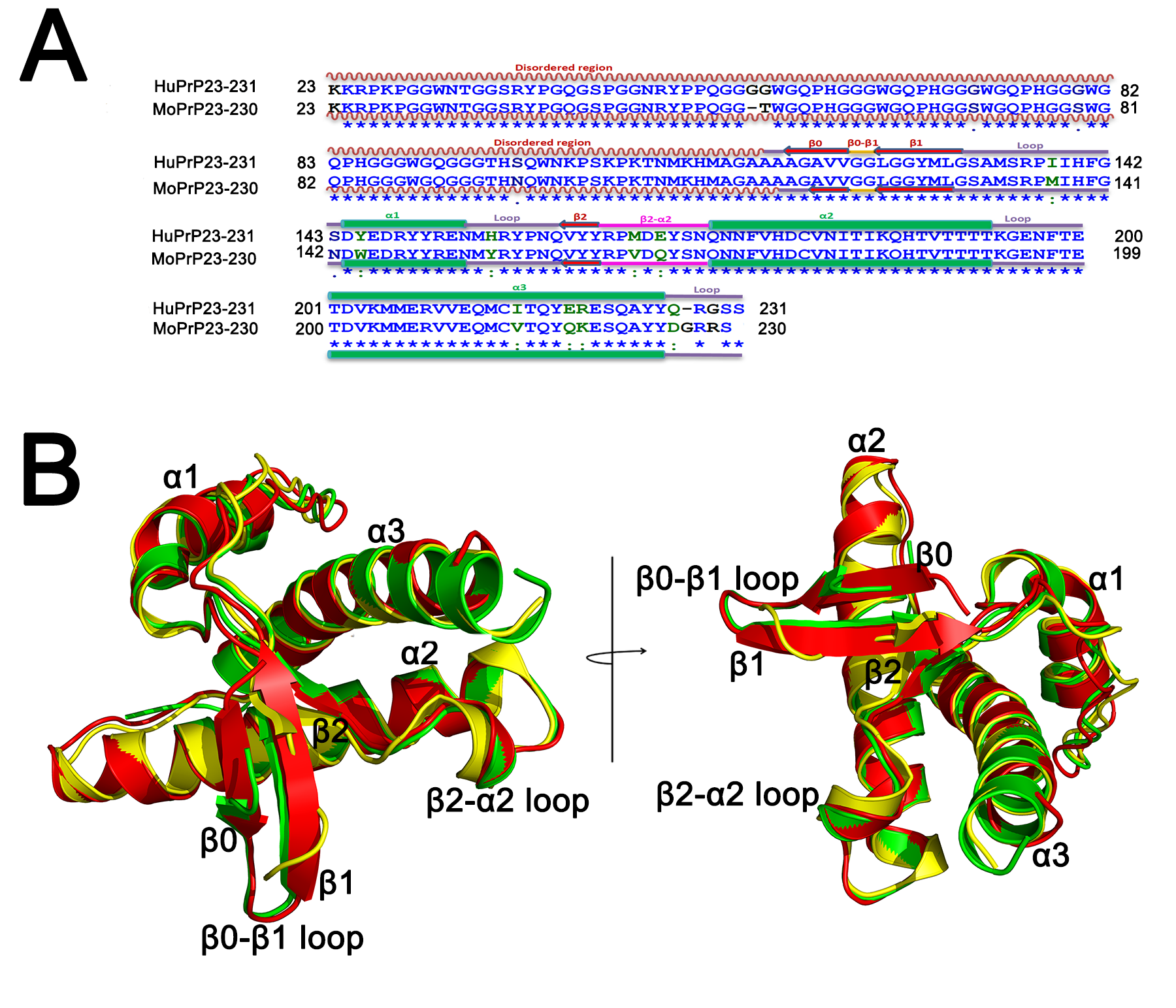

Supplement: S2 Fig — (A) The amino-acid sequence alignment of representative PrPs showing different in the structural elements between MoPrP and HuPrP. (B) Structural comparisons of MoPrP(89–230) (MoPrP is depicted in green) with the HuPrP23-231 (PDB 4KML, X-ray) in red and MoPrP(124–230) (PDB 4H88, X-ray) in yellow. (TIF) [file ppat.1008139.s002.tif]

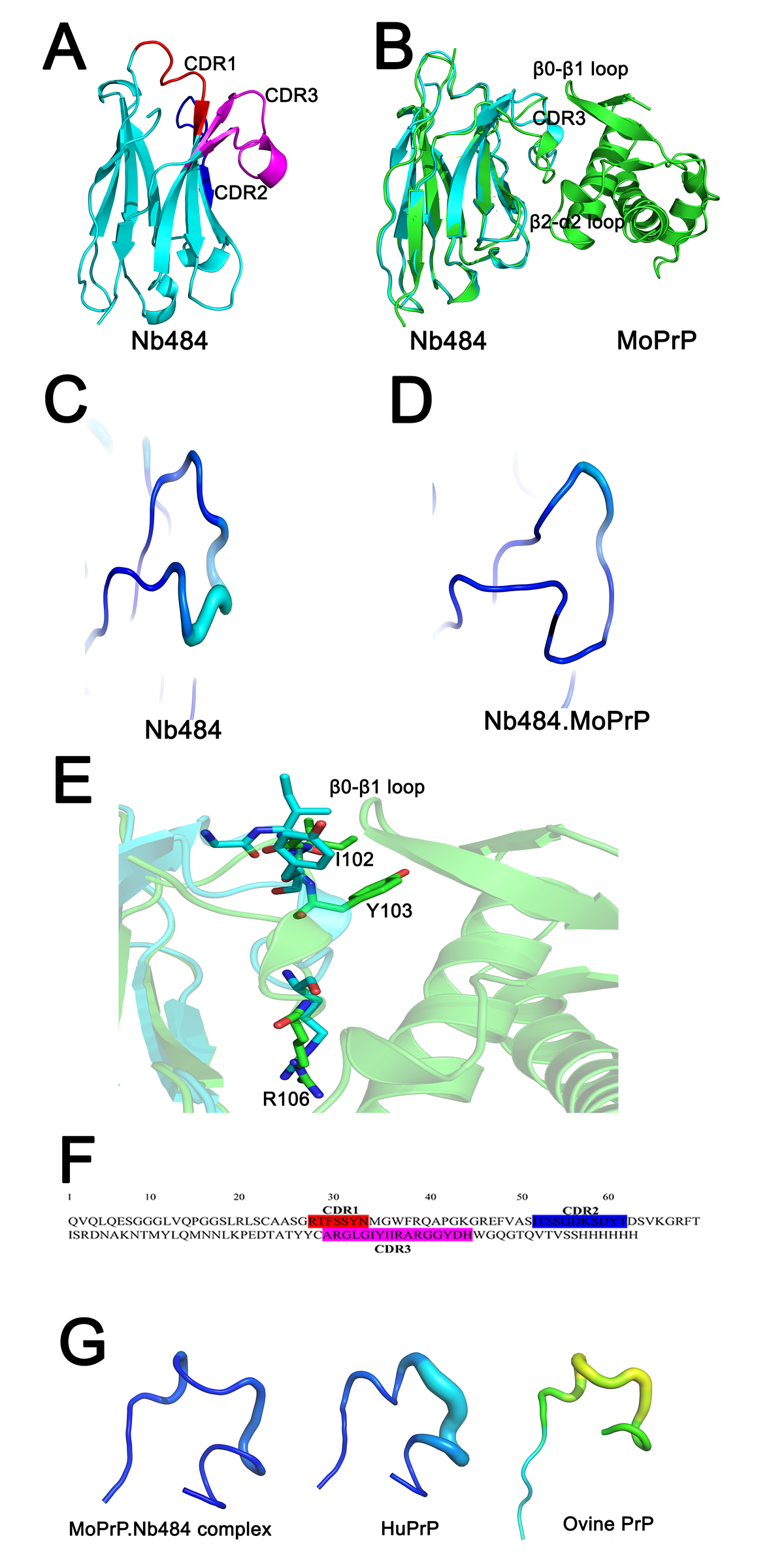

Supplement: S3 Fig — (A) Cartoon representation of X-rays structure of Nb484 alone showing its CDRs. The CDR1 region is shown in red, the CDR2 region in blue and CDR3 in pink. (B) Structural comparison of unbound Nb484 (cyan) with the same nanobody bound to MoPrP(89–230) in green. (C) Structural flexibility of the CDR3 in Nb484 alone and (D) the MoPrP•Nb484 complex illustrating the thermal parameter distributions in the CDR3 using the B-factor putty tube representation as implemented in PyMol. (E) The conformational changes of the interacting residues of the Nb484 with MoPrP. (F) The amino acid sequence of Nb484 with CDRs according to IMGT indicated in color. (G) Structural flexibility of the β2-α2 loop in X-ray structures. Illustration of the thermal parameter distribution in the β2-α2 loops of the MoPrP(89–230)•Nb484 complex (this study), HuPrP(23–231) alone (PDB 3HAK) and Ovine PrP(114–234) (PDB 1TPX) using the B-factor putty tube representation as implemented in PyMol. (TIF) [file ppat.1008139.s003.tif]

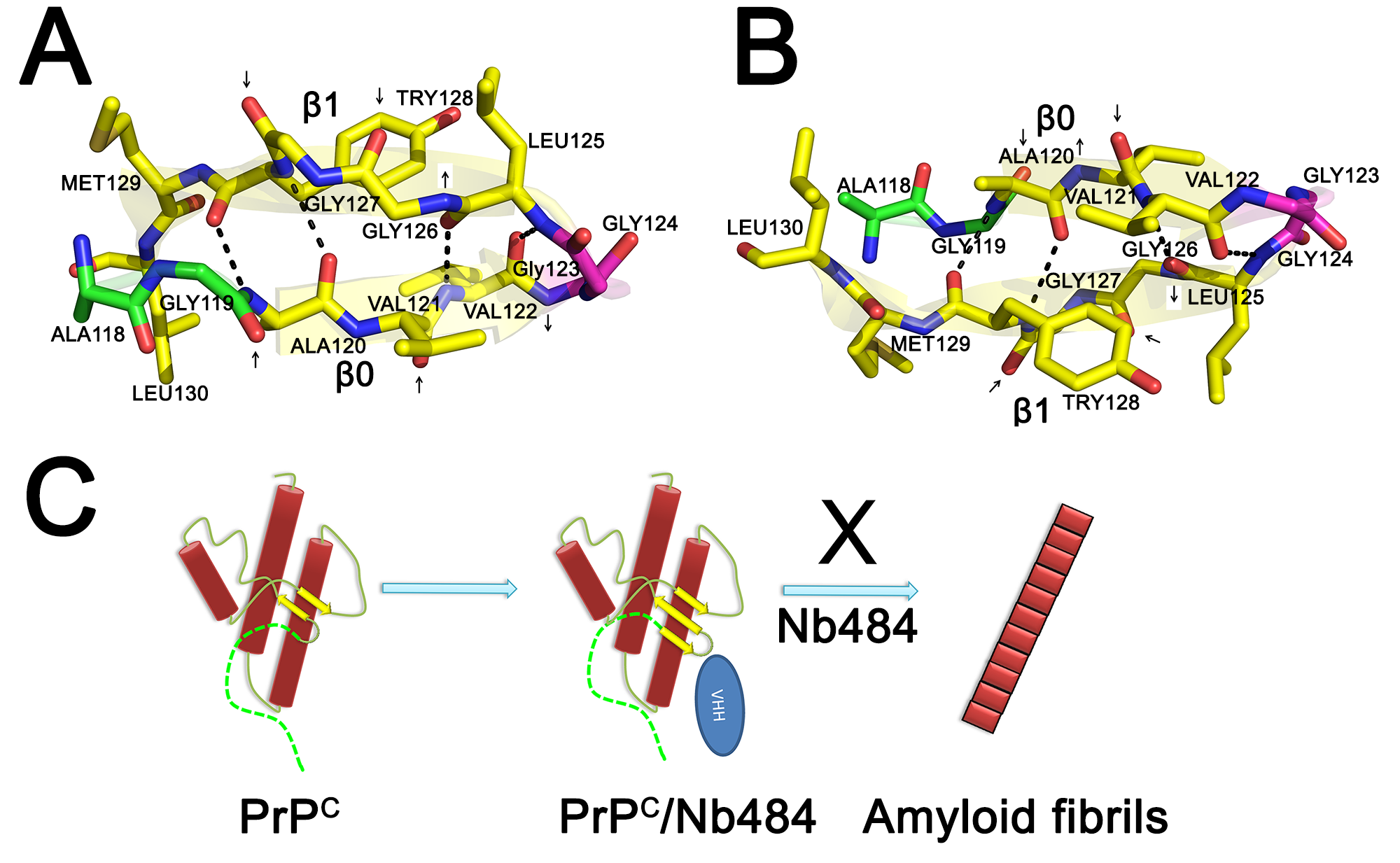

Supplement: S4 Fig — The backbone donor and acceptor sites exposed to solvent are indicated by arrows. (A) Solvent exposed face of the β0-β1 hairpin. (B) Solvent protected face of the hairpin. (C) Model of conformation changes from cellular PrPC to infectious PrPSc, PrPC converted to β-sheet form then followed by self-assembling into amyloid fiber by un-known mechanism. The formation β0-β1 hairpin shows backbone H-bond donor and acceptor sites to solvent. These sites can serve as a structural nucleus for the growth of amyloid fibrils, which can be inhibited by binding of Nb484. (TIF) [file ppat.1008139.s004.tif]

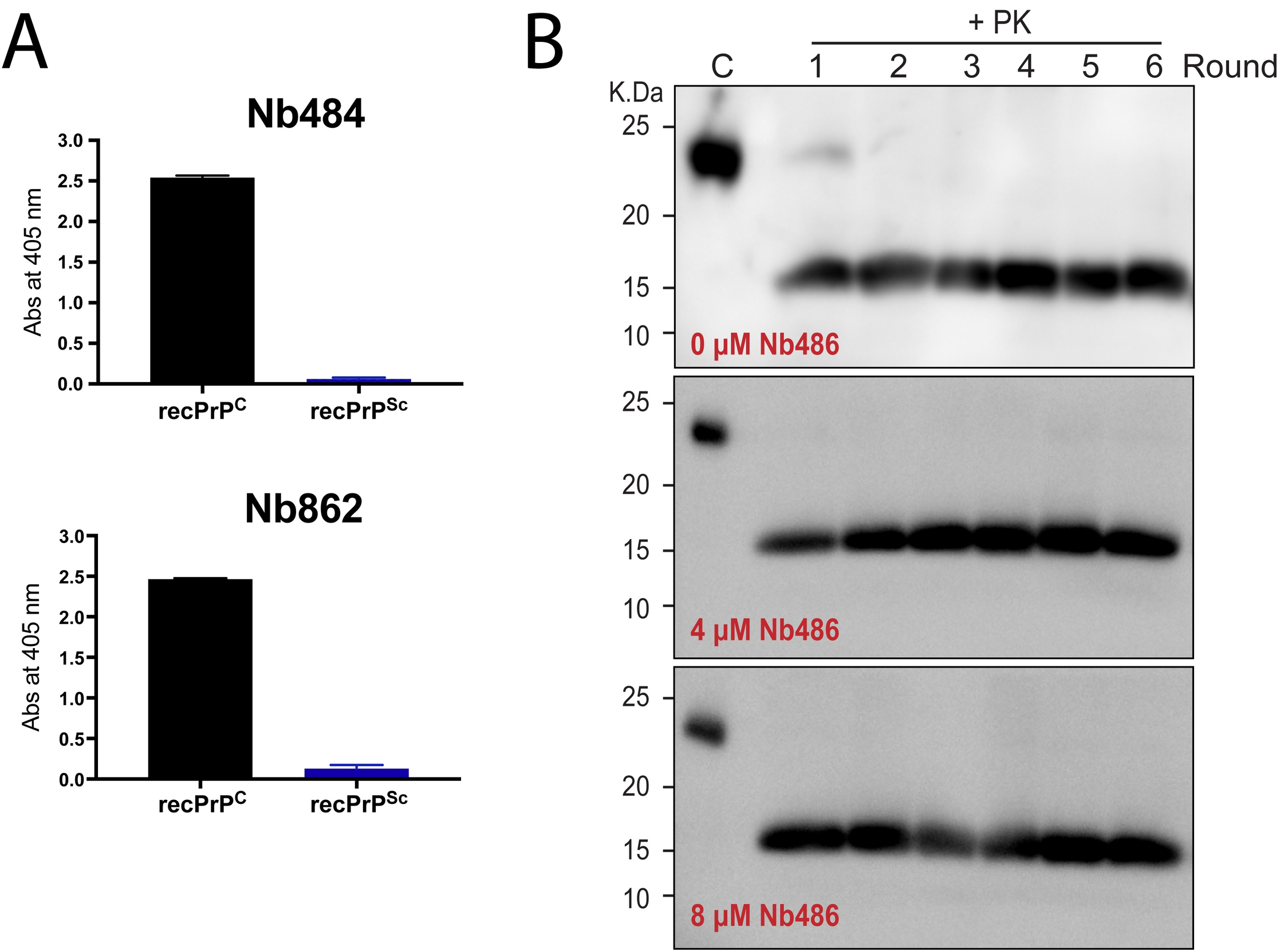

Supplement: S5 Fig — (A) ELISA assay of Nb484 or Nb862 against recombinant PrPC (23–230) and PrPSc (+PK). The assay was monitored by measuring the absorbance at 405 nm. (B) Effect of Nb486 on the prion amplification. Inhibition of prion propagation by different concentrations (4 and 8 μM) of Nb486 in Protein Misfolding Cyclic amplification (PMCA) for six consecutive rounds. Nb486 has low binding affinity to recPrP, show no effect on the prion propagation in PMCA (S3 Table). (TIF) [file ppat.1008139.s005.tif]

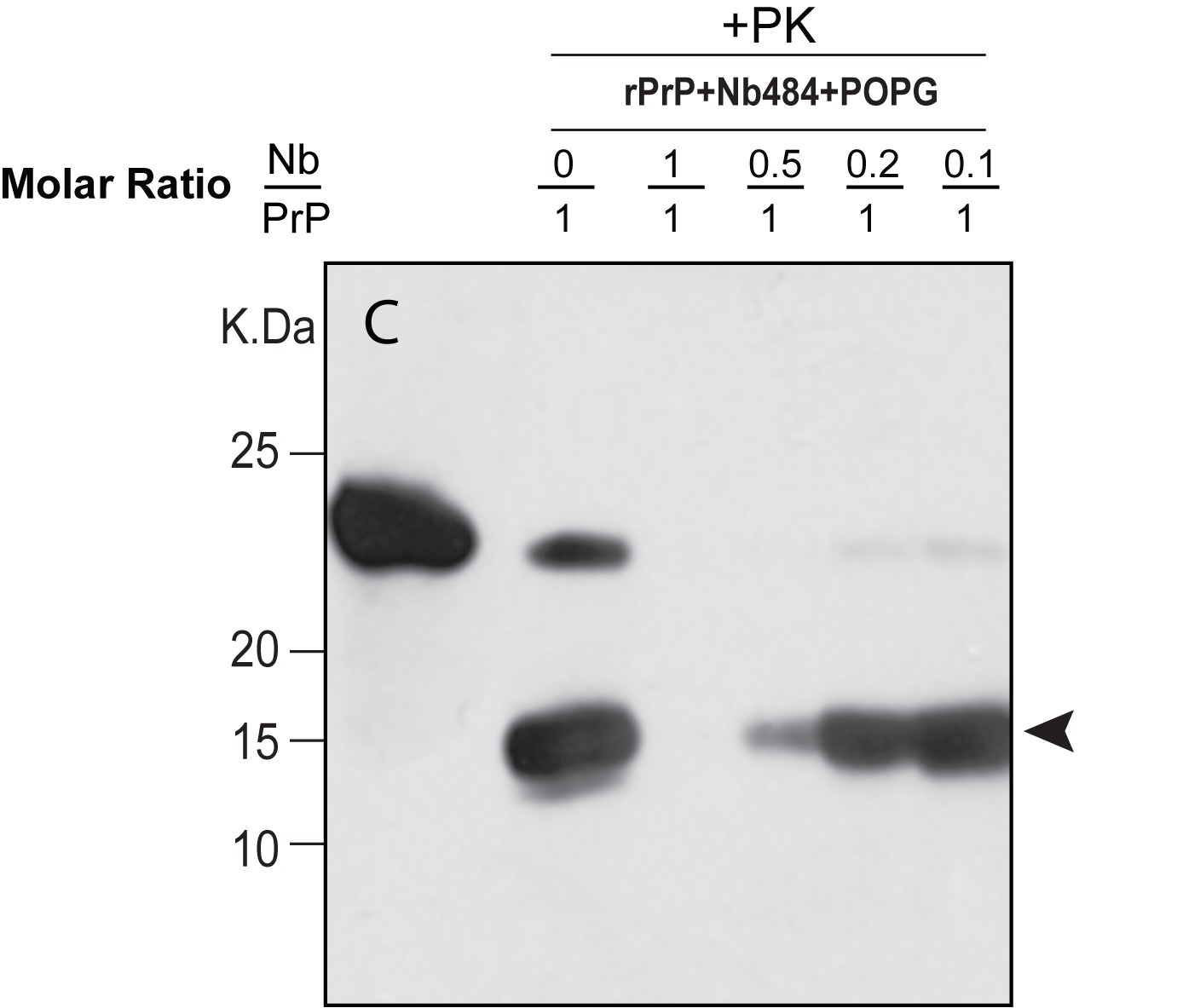

Supplement: S6 Fig — rPrP was incubated with Nb484 at different molar ratios (Nb484:rPrP = 0, 1, 0.5, 0.2 or 0.1:1) before mixed with POPG. POPG-induced PK-resistance was completely inhibited at Nb484:rPrP = 1:1. rPrP: MoPrP(23–230). (TIF) [file ppat.1008139.s006.tif]

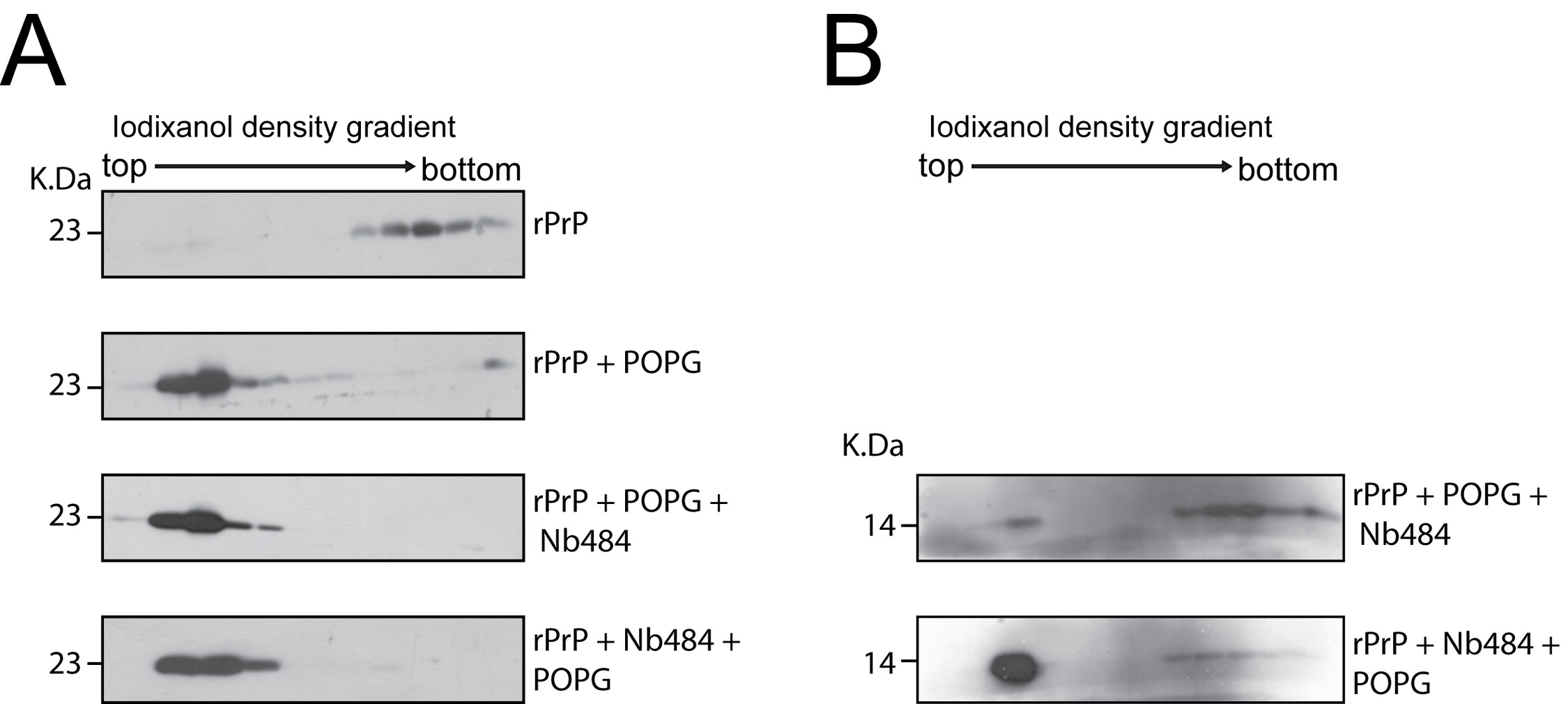

Supplement: S7 Fig — (A) Iodixanol density gradient analysis of rPrP, rPrP + POPG, rPrP + POPG + Nb484 and rPrP + Nb484 + POPG using POM1 antibody. (B) Iodixanol density gradient analysis of rPrP + POPG + Nb484 and rPrP + Nb484 + POPG using Anti-histidine antibody to detect Nb484. rPrP: MoPrP(23–230). (TIF) [file ppat.1008139.s007.tif]

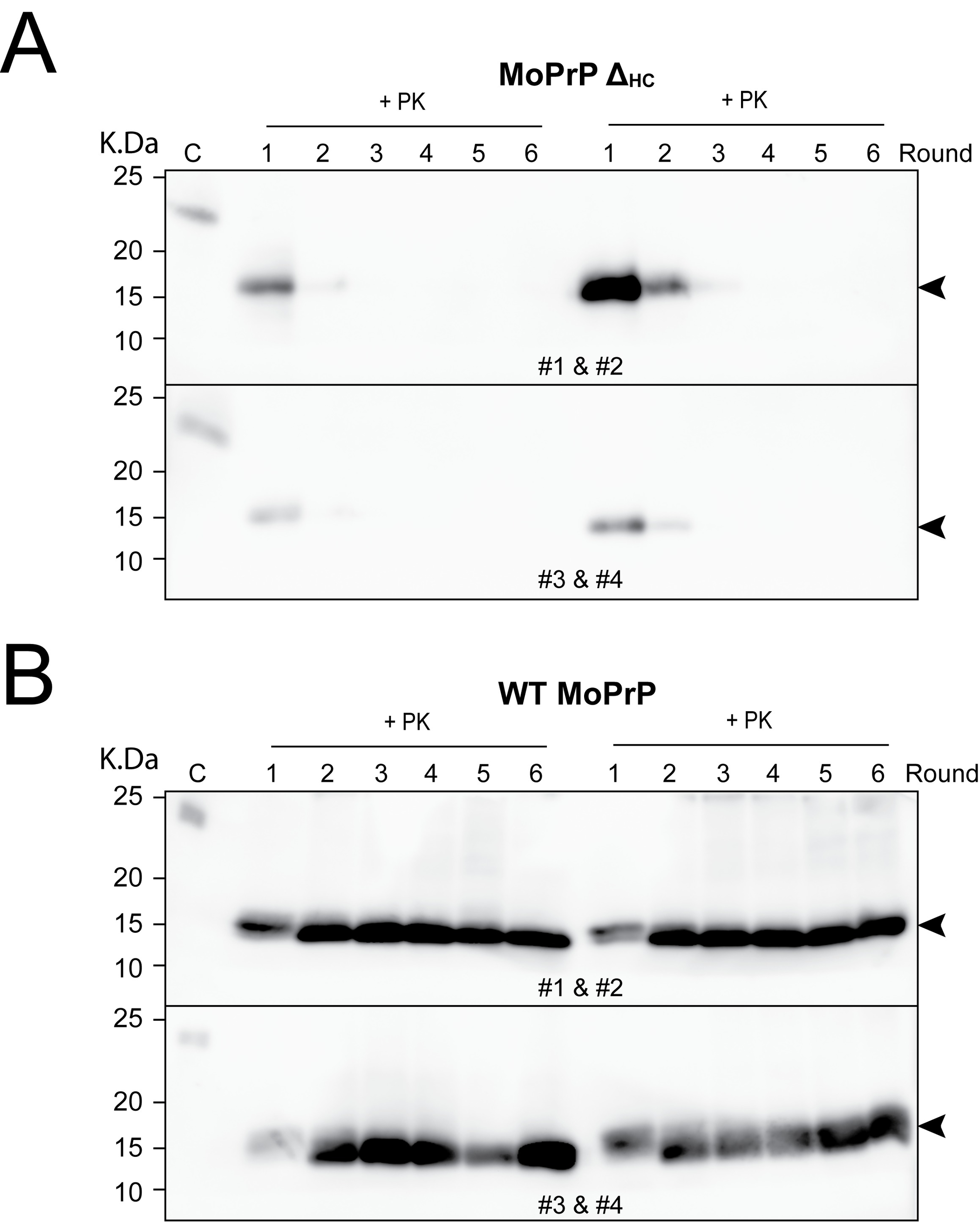

Supplement: S8 Fig — (A) MoPrPΔHC was used as the substrate and seeded by rec-prion seeds in PMCA for six consecutive rounds. Four replicates of MoPrPΔHC PMCA were performed (B) Full-length mouse PrP (WT MoPrP) was used as a positive control for rec-prionPMCA (four replicates). (TIF) [file ppat.1008139.s008.tif]

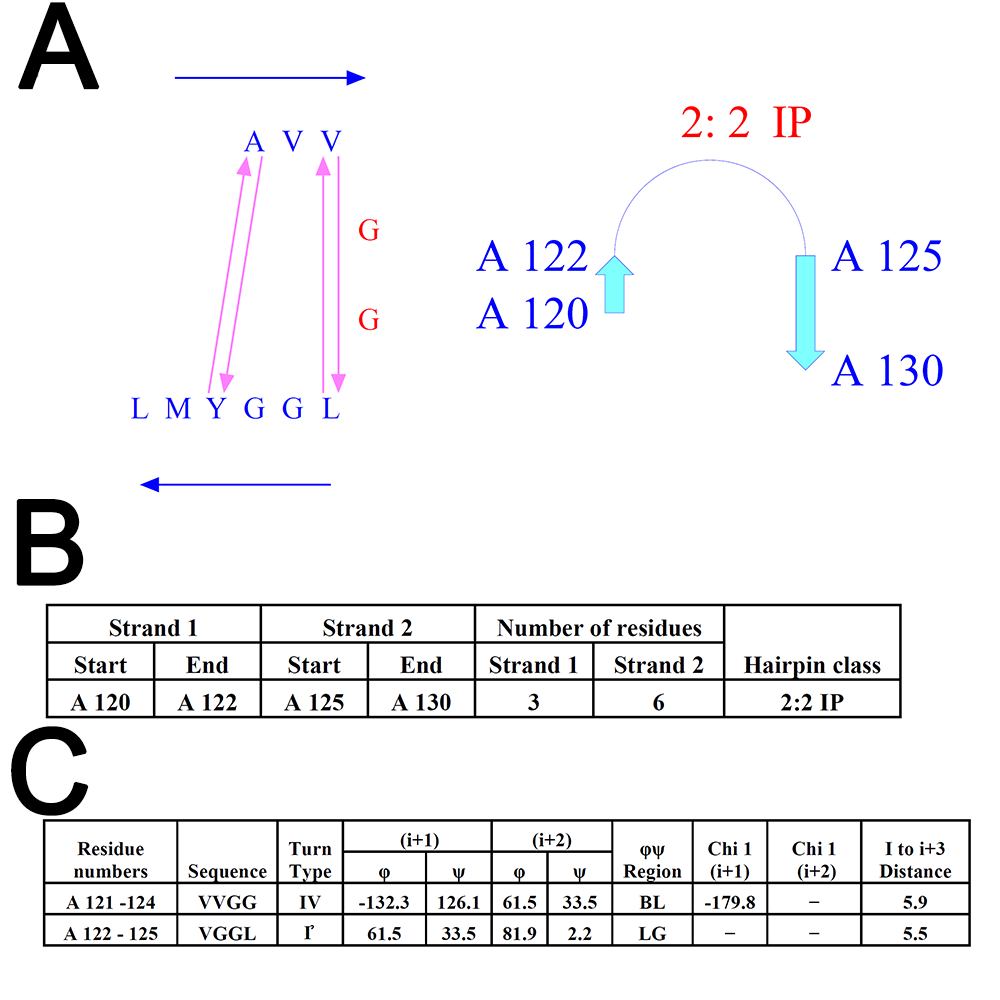

Supplement: S9 Fig — (A) Schematic representation of the β0-β1 β-hairpin. Residues of the antiparallel β-strands are indicated in blue. Hydrogen bonds are represented by pink arrows. (B) Statistics of the 2:2 IP type β0-β1 β-hairpin. (C) Statistics of the β-turn. (TIF) [file ppat.1008139.s009.tif]

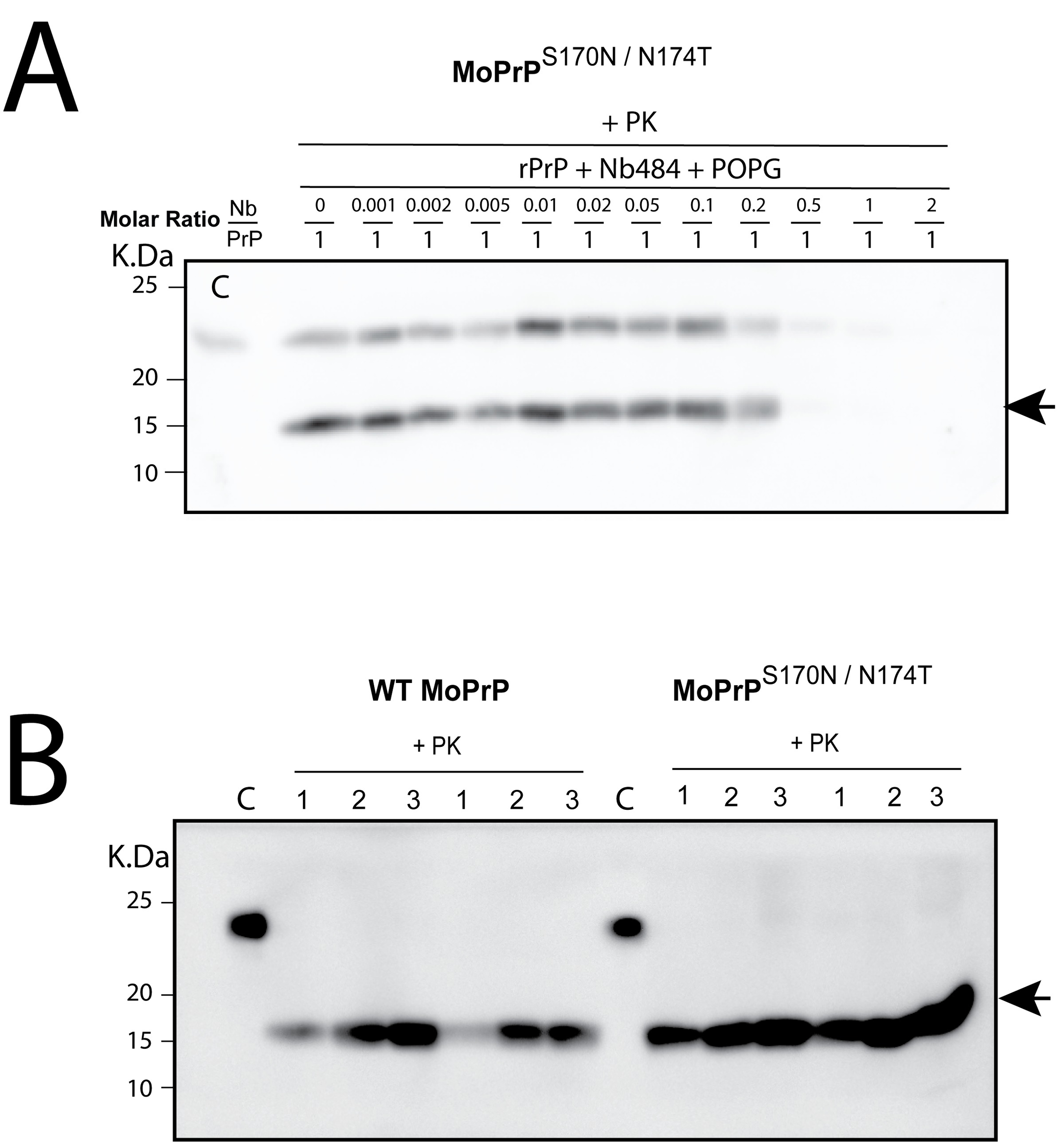

Supplement: S10 Fig — (A) MoPrPS170N/N174T was incubated Nb484 at different molar ratios before mixed with POPG. PK-resistant MoPrPS170N/N174T was detected using POM1 antibody. (B) Full-length WT MoPrP and MoPrPS170N/N174T mutant were used as the substrates and seeded by rec-prion seeds in PMCA for three consecutive rounds. Two replicates for each substrate. (TIF) [file ppat.1008139.s010.tif]

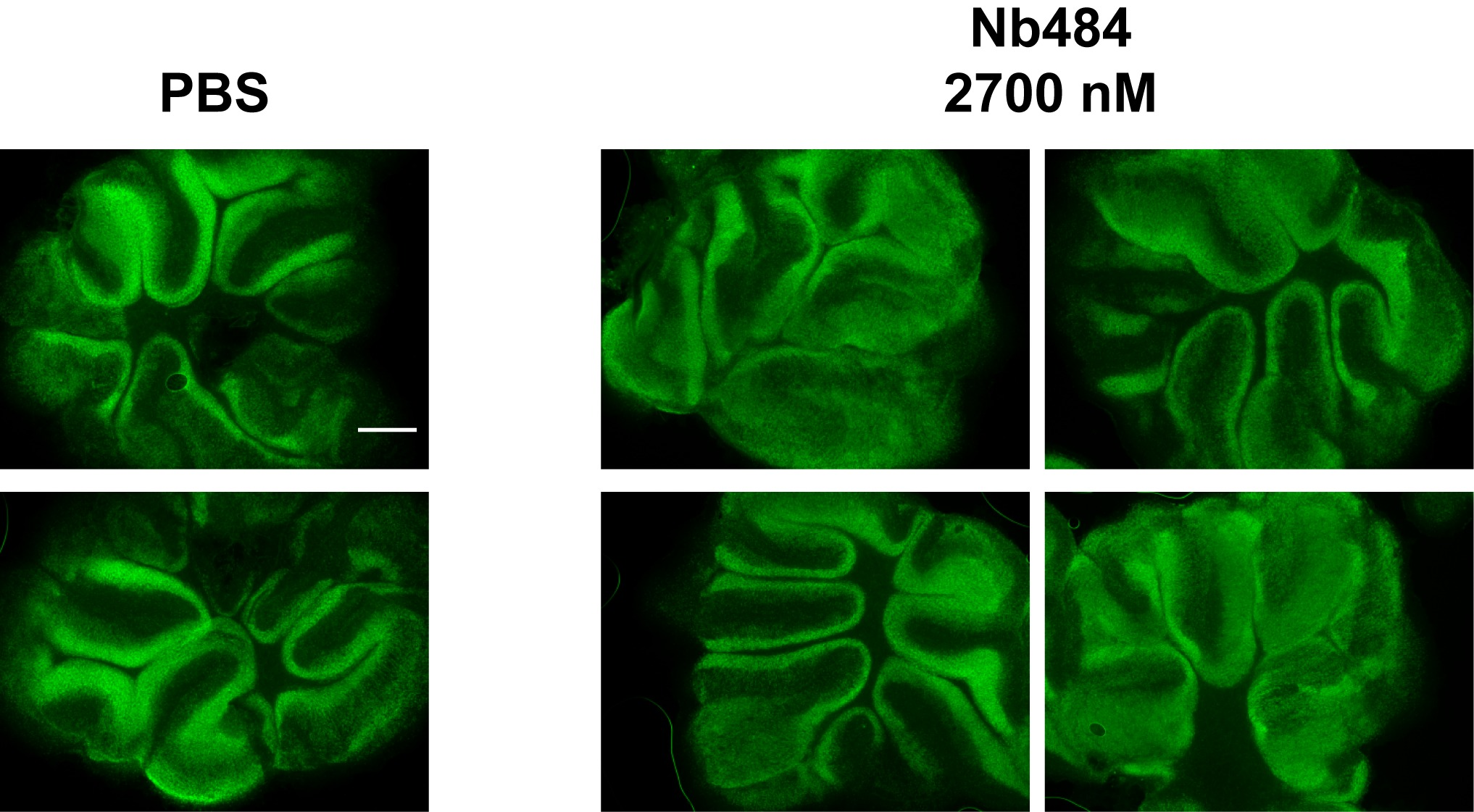

Supplement: S11 Fig — Cerebellar organotypic slices are healthy when incubated with Nb484 at concentration of 2700 nM, similar to PBS treated slices. The white bar is 100 μm. (TIF) [file ppat.1008139.s011.tif]
