# Supplementary material for: Structural evidence for the critical role of the prion protein hydrophobic region in forming an infectious prion
Source: PLoS Pathog. 2019 Dec 9;15(12):e1008139. doi: 10.1371/journal.ppat.1008139 (PMC6922452; doi:10.1371/journal.ppat.1008139)
Supplement: S1 Table — (DOCX) [file ppat.1008139.s012.docx]

**S1 Table**. Intermolecular interactions between MoPrP(89-230) and Nb484 in the crystal structure of the complex.

| **MoPrP** | **Secondary structures** | **Nb484** | **Secondary structures** | | **Distance (Å)** | **Interactions** | |
| --- | --- | --- | --- | --- | --- | --- | --- |
| GLY123 (O) | β0-β1 loop | ILE102 (N) | CDR3 | | 2.82 | Hydrogen Bonds | |
| GLN168 (O) | β2-α2 loop | ARG108 (N) | CDR3 | | 2.95 | Hydrogen Bonds | |
| ASP178 (OD1) | α2 | ARG106 (N) | CDR3 | | 2.81 | Hydrogen Bonds | |
| ASP178 (OD2) | α2 | ARG106 (N) | CDR3 | | 3.28 | Hydrogen Bonds | |
| ASP178 (OD2) | α2 | ALA107 (N) | CDR3 | | 3.03 | Hydrogen Bonds | |
| ASP167 (O) | β2-α2 loop | ARG108 (NH1) | CDR3 | | 3.1 | Hydrogen Bonds | |
| GLN168 (OE1) | β2-α2 loop | GLY109 (N) | CDR3 | | 3.02 | Hydrogen Bonds | |
| ASN173 (ND2) | α2 | ASP62 (OD2) | CDR2 | | 3.22 | Hydrogen Bonds | |
| ASN174 (OD1) | α2 | THR61 (OG1) | CDR2 | | 3.09 | Hydrogen Bonds | |
| ASN174 (ND2) | α2 | ARG106 (NH1) | CDR3 | | 2.85 | Hydrogen Bonds | |
| HIS177 (NE2) | α2 | ARG106 (NH1) | CDR3 | | 3.32 | Hydrogen Bonds | |
| ASP167 (OD1) | β2-α2 loop | ARG108 (NH1) | CDR3 | | 2.87 | Hydrogen Bonds | |
| ARG164 (NH1) | β2-α2 loop | GLY109 (O) | CDR3 | | 3.81 | Hydrogen Bonds | |
| ASP167 (OD1) | β2-α2 loop | ARG108 (NH2) | CDR3 | | 2.95 | Hydrogen Bonds | |
| GLY123 (CA) | β0-β1 loop | ILE102 (CG1) | CDR3 | | 3.73 | Hydrophobic interaction | |
| GLY123 (CA) | β0-β1 loop | TYR103 (CE2) | CDR3 | | 3.79 | Hydrophobic interaction | |
| GLY123 (O) | β0-β1 loop | GLY101 (CA) | CDR3 | | 3.12 | Hydrophobic interaction | |
| GLY123 (O) | β0-β1 loop | GLY101 (C) | CDR3 | | 3.41 | Hydrophobic interaction | |
| GLY 123 (O) | β0-β1 loop | ILE102 (N) | CDR3 | | 2.82 | Hydrophobic interaction | |
| GLY123 (O) | β0-β1 loop | ILE102 (CG1) | CDR3 | | 3.57 | Hydrophobic interaction | |
| LEU125 (CD1) | β0-β1 loop | LEU100 (CB) | CDR3 | | 3.84 | Hydrophobic interaction | |
| LEU125 (CD1) | β0-β1 loop | GLY109 (O) | CDR3 | | 3.72 | Hydrophobic interaction | |
| LEU125 (CD2) | β0-β1 loop | ILE105 (CD1) | CDR3 | | 3.73 | Hydrophobic interaction | |
| TYR128 (OH) | β1 | ILE105 (CD1) | CDR3 | | 3.62 | Hydrophobic interaction | |
| TYR128 (OH) | β1 | ILE105 (CG2) | CDR3 | | 3.82 | Hydrophobic interaction | |
| ARG164 (CD) | β2-α2 loop | ILE105 (CD1) | CDR3 | | 3.78 | Hydrophobic interaction | |
| ARG164 (CD) | β2-α2 loop | ALA107 (CB) | CDR3 | | 3.54 | Hydrophobic interaction | |
| ARG164 (NH1) | β2-α2 loop | GLY109 (O) | CDR3 | | 3.82 | Hydrophobic interaction | |
| ASP167 (OD1) | β2-α2 loop | ARG108 (CZ) | CDR3 | | 3.34 | Hydrophobic interaction | |
| ASP167 (OD1) | β2-α2 loop | ARG108 (NH1 | CDR3 | | 2.87 | Hydrophobic interaction | |
| ASP167 (OD1) | β2-α2 loop | ARG108 (NH2) | CDR3 | | 2.95 | Hydrophobic interaction | |
| ASP167 (C) | β2-α2 loop | ARG108 (NH1) | CDR3 | | 3.64 | Hydrophobic interaction | |
| ASP167 (O) | β2-α2 loop | ARG108 (NH1) | CDR3 | | 3.1 | Hydrophobic interaction | |
| GLN168 (N) | β2-α2 loop | ARG108 (NH1) | CDR3 | | 3.85 | Hydrophobic interaction | |
| GLN168 (CA) | β2-α2 loop | ARG108 (NH1) | CDR3 | | 3.67 | Hydrophobic interaction | |
| GLN168 (CB) | β2-α2 loop | ARG108 (N) | CDR3 | | 3.81 | Hydrophobic interaction | |
| GLN168 (CG) | β2-α2 loop | ARG108 (CZ) | CDR3 | | 3.87 | Hydrophobic interaction | |
| GLN168 (CG) | β2-α2 loop | ARG108 (NH2) | CDR3 | | 3.61 | Hydrophobic interaction | |
| GLN168 (CD) | β2-α2 loop | GLY109 (N) | CDR3 | | 3.72 | Hydrophobic interaction | |
| GLN168 (OE1) | β2-α2 loop | ALA107 (CB) | CDR3 | | 3.47 | Hydrophobic interaction | |
| GLN168 (OE1) | β2-α2 loop | GLY109 (N) | CDR3 | | 3.02 | Hydrophobic interaction | |
| GLN168 (OE1) | β2-α2 loop | GLY109 (CA) | CDR3 | | 3.25 | Hydrophobic interaction | |
| GLN168 (O) | β2-α2 loop | ALA107 (CA) | CDR3 | | 3.42 | Hydrophobic interaction | |
| GLN168 (O) | β2-α2 loop | ALA107 (C) | CDR3 | | 3.67 | Hydrophobic interaction | |
| GLN168 (O) | β2-α2 loop | ARG108 (N) | CDR3 | | 2.95 | Hydrophobic interaction | |
| TYR169 (CE1) | β2-α2 loop | ALA107 (N) | CDR3 | | 3.54 | Hydrophobic interaction | |
| TYR169 (CE1) | β2-α2 loop | ALA107 (CA) | CDR3 | | 3.6 | Hydrophobic interaction | |
| TYR169 (CE1) | β2-α2 loop | ALA107 (CB) | CDR3 | | 3.77 | Hydrophobic interaction | |
| TYR169 (CZ) | β2-α2 loop | ALA107 (CB) | CDR3 | | 3.56 | Hydrophobic interaction | |
| TYR169 (OH) | β2-α2 loop | ALA107 (CB) | CDR3 | | 3.42 | Hydrophobic interaction | |
| ASN173 (CG) | α2 | ASP62 (OD2) | CDR2 | | 3.86 | Hydrophobic interaction | |
| ASN173 (ND2) | α2 | ASP62 (CG) | CDR2 | | 3.7 | Hydrophobic interaction | |
| ASN173 (ND2) | α2 | ASP62 (OD2) | CDR2 | | 3.22 | Hydrophobic interaction | |
| ASN173 (ND2) | α2 | ARG106 (NH1) | CDR3 | | 3.82 | Hydrophobic interaction | |
| ASN174 (CG) | α2 | THR61 (OG1) | CDR2 | | 3.8 | Hydrophobic interaction | |
| ASN174 (OD1) | α2 | THR61 (OG1) | CDR2 | | 3.09 | Hydrophobic interaction | |
| ASN174 (ND2) | α2 | THR61 (OG1) | CDR2 | | 3.69 | Hydrophobic interaction | |
| ASN174 (ND2) | α2 | ARG106 (CD) | CDR3 | | 3.31 | Hydrophobic interaction | |
| ASN174 (ND2) | α2 | ARG106 (CZ) | CDR3 | | 3.81 | Hydrophobic interaction | |
| ASN174 (ND2) | α2 | ARG106 (NH1) | CDR3 | | 2.85 | Hydrophobic interaction | |
| HIS177 (CB) | α2 | ARG106 (NH1) | CDR3 | | 3.64 | Hydrophobic interaction | |
| HIS177 (CG) | α2 | ARG106 (NE) | CDR3 | | 3.82 | Hydrophobic interaction | |
| HIS177 (CG) | α2 | ARG106 (CZ) | CDR3 | | 3.4 | Hydrophobic interaction | |
| HIS177 (CG) | α2 | ARG106 (NH1) | CDR3 | | 3.54 | Hydrophobic interaction | |
| HIS177 (CG) | α2 | ARG106 (NH2) | CDR3 | | 3.62 | Hydrophobic interaction | |
| HIS177 (ND1) | α2 | ARG106 (NE) | CDR3 | | 3.64 | Hydrophobic interaction | |
| HIS177 (ND1) | α2 | ARG106 (CZ) | CDR3 | | 3.59 | Hydrophobic interaction | |
| HIS177 (ND1) | α2 | ARG106 (NH2) | CDR3 | | 3.69 | Hydrophobic interaction | |
| HIS177 (CE1) | α2 | ARG106 (CZ) | CDR3 | | 3.81 | Hydrophobic interaction | |
| HIS177 (CE1) | α2 | ARG106 (NH2) | CDR3 | | 3.44 | Hydrophobic interaction | |
| HIS177 (NE2) | α2 | ARG106 (CZ) | CDR3 | | 3.89 | Hydrophobic interaction | |
| HIS177 (NE2) | α2 | ARG106 (NH2) | CDR3 | | 3.32 | Hydrophobic interaction | |
| HIS177 (CD2) | α2 | ARG106 (CZ) | CDR3 | | 3.62 | Hydrophobic interaction | |
| HIS177 (CD2) | α2 | ARG106 (NH1) | CDR3 | | 3.78 | Hydrophobic interaction | |
| HIS177 (CD2) | α2 | ARG106 (NH2) | CDR3 | | 3.4 | Hydrophobic interaction | |
| ASP178 (CG) | α2 | ILE105 (CB) | CDR3 | | 3.68 | Hydrophobic interaction | |
| ASP178 (CG) | α2 | ARG106 (N) | CDR3 | | 3.36 | Hydrophobic interaction | |
| ASP178 (OD1) | α2 | ILE105 (CA) | CDR3 | | 3.62 | Hydrophobic interaction | |
| ASP178 (OD1) | α2 | ILE105 (CB) | CDR3 | | 3.49 | Hydrophobic interaction | |
| ASP178 (OD1) | α2 | ILE105 (C) | CDR3 | | 3.59 | Hydrophobic interaction | |
| ASP178 (OD1) | α2 | ARG106 (N) | CDR3 | | 2.81 | Hydrophobic interaction | |
| ASP178 (OD1) | α2 | ARG106 (CA) | CDR3 | | 3.68 | Hydrophobic interaction | |
| ASP178 (OD1) | α2 | ARG106 (CB) | CDR3 | | 3.57 | Hydrophobic interaction | |
| ASP178 (OD1) | α2 | ARG106 (CG) | CDR3 | | 3.77 | Hydrophobic interaction | |
| ASP178 (OD2) | α2 | ILE105 (CB) | CDR3 | | 3.52 | Hydrophobic interaction | |
| ASP178 (OD2) | α2 | ILE105 (CD1) | CDR3 | | 3.86 | Hydrophobic interaction | |
| ASP178 (OD2) | α2 | ARG106 (N) | CDR3 | | 3.28 | Hydrophobic interaction | |
| ASP178 (OD2) | α2 | ARG106 (CA) | CDR3 | | 3.89 | Hydrophobic interaction | |
| ASP178 (OD2) | α2 | ARG106 (CA) | CDR3 | | 3.81 | Hydrophobic interaction | |
| ASP178 (OD2) | α2 | ALA107 (N) | CDR3 | | 3.03 | Hydrophobic interaction | |
| ASP178 (OD2) | α2 | ALA107 (CA) | CDR3 | | 3.78 | Hydrophobic interaction | |
| ASP178 (OD2) | α2 | ALA107 (CB) | CDR3 | | 3.52 | Hydrophobic interaction | |
| ILE182 (CD1) | α2 | ILE105 (CG2) | CDR3 | | 3.8 | Hydrophobic interaction | |
| LYS185 (CD) | α2 | TYR103 (CG) | CDR3 | | 3.67 | Hydrophobic interaction | |
| LYS185 (CD) | α2 | TYR103 (CD1) | CDR3 | | 3.48 | Hydrophobic interaction | |
| LYS185 (CD) | α2 | TYR103 (CE1) | CDR3 | | 3.53 | Hydrophobic interaction | |
| LYS185 (CD) | α2 | TYR103 (CZ) | CDR3 | | 3.72 | Hydrophobic interaction | |
| LYS185 (CD) | α2 | TYR103 (CE2) | CDR3 | | 3.87 | Hydrophobic interaction | |
| LYS185 (CD) | α2 | TYR103 (CD2) | CDR3 | | 3.83 | Hydrophobic interaction | |
| LYS185 (CE) | α2 | TYR103 (CG) | CDR3 | | 3.84 | Hydrophobic interaction | |
| LYS185 (CE) | α2 | TYR103 (CD1) | CDR3 | | 3.25 | Hydrophobic interaction | |
| LYS185 (CE) | α2 | TYR103 (CE1) | CDR3 | | 3.52 | Hydrophobic interaction | |
| VAL189 (CG2) | α2 | TYR103 (OH) | CDR3 | | 3.79 | Hydrophobic interaction | |
| ASP167 | β2-α2 loop | ARG108 | CDR3 | 6 | | | Ionic Interactions |
| HIS177 | α2 | ASP59 | CDR2 | 6 | | | Ionic Interactions |
| LYS185 | α2 | TYR103 | CDR3 | 5.27 | | | Cation-Pi Interactions |
