# Supplementary material for: Structural evidence for the critical role of the prion protein hydrophobic region in forming an infectious prion
Source: PLoS Pathog. 2019 Dec 9;15(12):e1008139. doi: 10.1371/journal.ppat.1008139 (PMC6922452; doi:10.1371/journal.ppat.1008139)
Supplement: S2 Table — Nb862 was generated from Llamas immunized with MoPrPSc and screened against MoPrP (23–230). Nb486 was generated and screened from Llamas immunized with recombinant MoPrP(23–230). (DOCX) [file ppat.1008139.s013.docx]

**S2 Table**. Binding kinetics of Nb862 and Nb486 for MoPrP(23-230). Nb862 was generated from Llama immunized with MoPrP^Sc^ and screened against MoPrP (23-230). Nb486 was generated and screened from Llama immunized with recombinant MoPrP(23-230).

| **Nanobodies (No)** | **antigen** | **KD (M)** | **K_on_ (1/Ms)** | **K_off_ (1/s)** | **Immunization** |
| --- | --- | --- | --- | --- | --- |
| Nb862 | MoPrP(23-230) | 1,58E-10 | 1,30E+03 | 2,06E-07 | MoPrP^Sc^ |
| Nb486 | MoPrP(23-230) | 1,07E-06 | 4,26E+03 | 4,56E-03 | MoPrP(23-231) |
