# Supplementary material for: Structural evidence for the critical role of the prion protein hydrophobic region in forming an infectious prion
Source: PLoS Pathog. 2019 Dec 9;15(12):e1008139. doi: 10.1371/journal.ppat.1008139 (PMC6922452; doi:10.1371/journal.ppat.1008139)
Supplement: S3 Table — (DOCX) [file ppat.1008139.s014.docx]

**S3 Table. Comparison of backbone superposition** root-mean-square deviation **values (RMSD, Å) of** PrP^C^s in complexes with antibody.

|  | MorP•Nb484 | MoPrP•POM1 Fab | HuPrP•ICSM18 Fab | OvPrP•VRQ14 Fab |
| --- | --- | --- | --- | --- |
| MoPrP•Nb484 | 0 | 0.436 | 0.677 | 0.884 |
| MoPrP•POM1 Fab | 0.436 | 0 | 0.695 | 0.65 |
| HuPrP•ICSM18 Fab | 0.677 | 0.695 | 0 | 0.654 |
| OvPrP•VRQ14 Fab | 0.884 | 0.65 | 0.654 | 0 |
